# Histone deacetylase 3 deletion in alveolar type 2 epithelial cells prevents bleomycin-induced pulmonary fibrosis

**DOI:** 10.1186/s13148-023-01588-5

**Published:** 2023-11-11

**Authors:** Rui Xiong, Boxin Geng, Wenyang Jiang, Yong Hu, Zhaoyu Hu, Bo Hao, Ning Li, Qing Geng

**Affiliations:** 1 Department of Thoracic Surgery, Renmin Hospital of Wuhan University, Jiefang Road 238, Wuhan, 430060 China; 2 Army Medical University, Chongqing, 430038 China; 3 Wuhan Rhegen Biotechnology Co., Ltd., Wuhan, 430073 China

**Keywords:** PF, HDAC3, EMT, Acetylation, GATA3

## Abstract

**Background:**

Epithelial mesenchymal transformation (EMT) in alveolar type 2 epithelial cells (AT2) is closely associated with pulmonary fibrosis (PF). Histone deacetylase 3 (HDAC3) is an important enzyme that regulates protein stability by modulating the acetylation level of non-histones. Here, we aimed to explore the potential role and regulatory mechanisms associated with HDAC3 in PF.

**Methods:**

We quantified HDAC3 expression both in lung tissues from patients with PF and from bleomycin (BLM)-treated mice. HDAC3 was also detected in TGF-β1-treated AT2. The mechanistic activity of HDAC3 in pulmonary fibrosis and EMT was also explored.

**Results:**

HDAC3 was highly expressed in lung tissues from patients with PF and bleomycin (BLM)-treated mice, especially in AT2. Lung tissues from AT2-specific HDAC3-deficient mice stimulated with BLM showed alleviative fibrosis and EMT. Upstream of HDAC3, TGF-β1/SMAD3 directly promoted HDAC3 transcription. Downstream of HDAC3, we also found that genetic or pharmacologic inhibition of HDAC3 inhibited GATA3 expression at the protein level rather than mRNA. Finally, we found that intraperitoneal administration of RGFP966, a selective inhibitor of HDAC3, could prevent mice from BLM-induced pulmonary fibrosis and EMT.

**Conclusion:**

TGF-β1/SMAD3 directly promoted the transcription of HDAC3, which aggravated EMT in AT2 and pulmonary fibrosis in mice via deacetylation of GATA3 and inhibition of its degradation. Our results suggest that targeting HDAC3 in AT2 may provide a new therapeutic target for the prevention of PF.

**Supplementary Information:**

The online version contains supplementary material available at 10.1186/s13148-023-01588-5.

## Introduction

Pulmonary fibrosis (PF) is often observed in the end stages of many lung diseases, but its pathogenesis remains unclear [1]. Idiopathic pulmonary fibrosis (IPF) is a common type of PF observed in clinical practice. Due to a lack of effective treatments, prognosis in patient with IPF is poor, and median survival time is only 3–5 years [2]. Therefore, there is an urgent need to elucidate the pathogenesis of IPF and establish effective treatments. Studies have shown that fibroblast foci (FF) are present in lung tissue from patients with IPF, comprising fibroblasts and myofibroblasts that are active in the extracellular matrix (ECM), which is the main pathological change observed in patients with IPF [3]. The repeated cycle of injury and repair in alveolar epithelial cells, composed of alveolar type I epithelial cells (AT1) and alveolar type II epithelial cells (AT2), also plays an important role in the pathology of IPF [4].

AT2 cells are a critical component of the respiratory barrier, which regulates the pulmonary immune system to protect the lungs from injury [5]. AT2 cells are an important source of FF following epithelial-mesenchymal transition (EMT) [6], which results from persistent cell damage and inflammation. Increasing evidence shows that EMT is one of the key pathological events in IPF [7]. When EMT occurs, cells undergo functional changes, dissociate from neighboring cells, and migrate to neighboring tissues [8]. In addition, the expression of epithelial cell markers such as E-cadherin, decreases, while the expression of interstitial cell markers, such as N-cadherin and vimentin, increases [9].

Histone deacetylases (HDACs) participate in the epigenetic regulation of gene transcription by regulating the acetylation levels of histone and non-histone proteins [10]. There are 11 HDAC family members in humans, which have been classified into three major subfamilies; class I containing HDACs 1, 2, 3 and 8, class II containing HDACs 4, 5, 6, 7, 9 and 10, and HDAC11 comprising a third distinct subtype [11]. In recent years, the functions of some HDACs in IPF have been elucidated. For instance, a previous study showed that knocking down HDAC4 attenuates TGF-β1-stimulated α-SMA expression in normal human lung fibroblasts [12], suggest that HDAC4 can modulate the production of ECM in lung myofibroblasts. Evidence suggests that HDAC2 is mainly involved in the chronic progression of pulmonary fibrosis, and inhibition of HDAC2 can alleviate bleomycin-induced pulmonary fibrosis in mice [13]. Recent data showed that HDAC3 promotes EMT and fibroblast migration under hypoxic conditions in A549 cells [14] and HDAC3 inhibition results in the acetylation and degradation of NICD1, thereby alleviating pulmonary fibrosis [15]. This further demonstrates that in addition to histone deacetylation, HDACs can deacetylate non-histone targets and co-regulate a wide range of biological and pathological processes [16]. However, the underlying mechanisms by which HDAC3 regulates EMT in AT2 cells have not yet been explored. Given the findings above, we speculate that HDAC3 may serve as a novel target for therapeutic intervention in bleomycin-induced EMT and pulmonary fibrosis.

In the present study, we found that HDAC3 was upregulated in lung tissues and AT2 cells from patients with IPF, and consistently detectable in lung tissues and AT2 cells from mice with BLM-induced pulmonary fibrosis. Moreover, HDAC3 deficiency in AT2 cells prevented mice from developing BLM-induced pulmonary fibrosis, which was accompanied by a marked reduction of EMT in AT2 cells from murine lung tissues. In terms of mechanisms, we found that TGF-β1/SMAD3 can directly promote HDAC3 transcription and further inhibit GATA3 acetylation and protein degradation, thus promoting EMT in BLM-induced AT2 cells. Consistent with this, intraperitoneal administration of the selective HDAC3 inhibitor, RGFP966, protected against BLM-induced pulmonary fibrosis in mice by targeting EMT in AT2 cells.

## Results

### IPF is characterized by increased HDAC3 expression in human lung tissues and AT2 cells

**Fig. 1:**
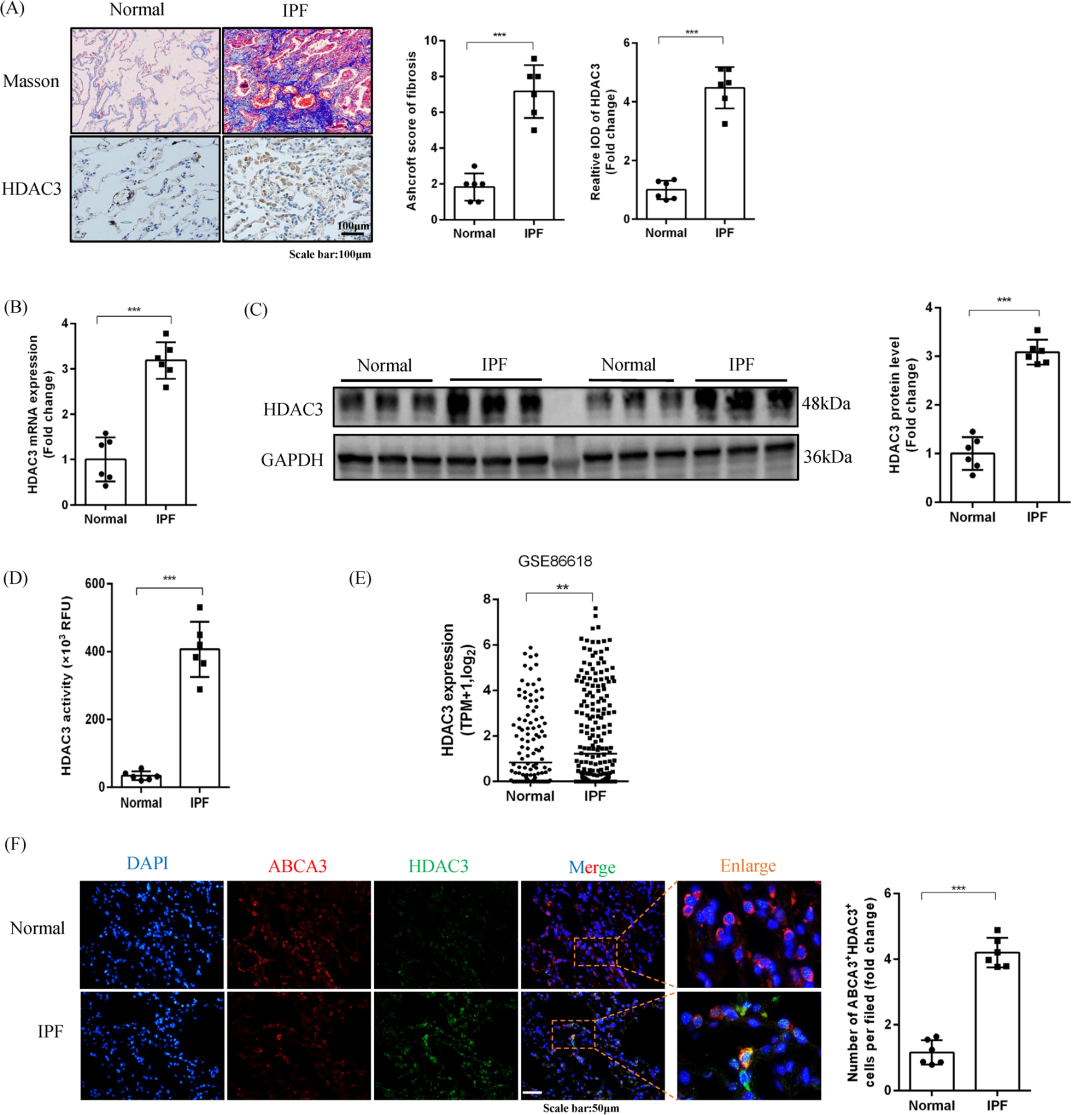
IPF is characterized by increased HDAC3 expression in human lung tissues and AT2. **A** Histological analyses showing masson staining and representative images showing immunohistochemical staining of HDAC3 in healthy controls and patients with IPF (× 200, n = 6). **B** HDAC3 mRNA quantification in patients with IPF vs healthy controls (n = 6). **C** Representative Western blot images and the corresponding quantitative results for HDAC3 in patients with IPF vs healthy controls (n = 6). **D** HDAC3 activity in lung tissues from patients with IPF vs healthy controls (n = 6). **E** Bioinformatics analysis of HDAC3 expression in AT2 cells from patients with IPF vs healthy controls (**F**). Representative images of HDAC3 and ABCA3 immunofluorescence colocalization staining and quantitation in lung tissues from patients with IPF and healthy controls (× 400, n = 6). (*P < 0.05, **P < 0.01, ***P < 0.001, ^ns^*P* > 0.05)

To explore the contribution of HDAC3 in IPF, we first measured the expression levels of HDAC3 in lung tissues from patients with IPF. As shown in Fig. 1A–C, compared with normal patients, both HDAC3 protein and mRNA expression were markedly increased in lung tissues from patients with IPF. Masson’s trichrome staining also showed significant deposition of collagen in the lung tissues of patients with IPF. In addition, patients with IPF showed higher HDAC3 activity in the lungs compared with controls (Fig. 1D).

We also found that HDAC3 expression was increased in both the nucleus and cytoplasm of the lungs from patients with IPF (Additional file 1: Figure S1A). Moreover, our bioinformatics analysis identified increased expression of HDAC3 in AT2 cells from patients with IPF (Fig. 1E), which was further supported by immunofluorescence staining (Fig. 1F). Furthermore, the fluorescence intensity of HDAC3 increased in both the nucleus and cytoplasm (Additional file 1: Figure S1B). These results suggested that HDAC3 expression in AT2 cells may participate in the development of IPF.

### HDAC3 is upregulated in lung tissues and AT2 cells from mice with BLM-induced pulmonary fibrosis

**Fig. 2:**
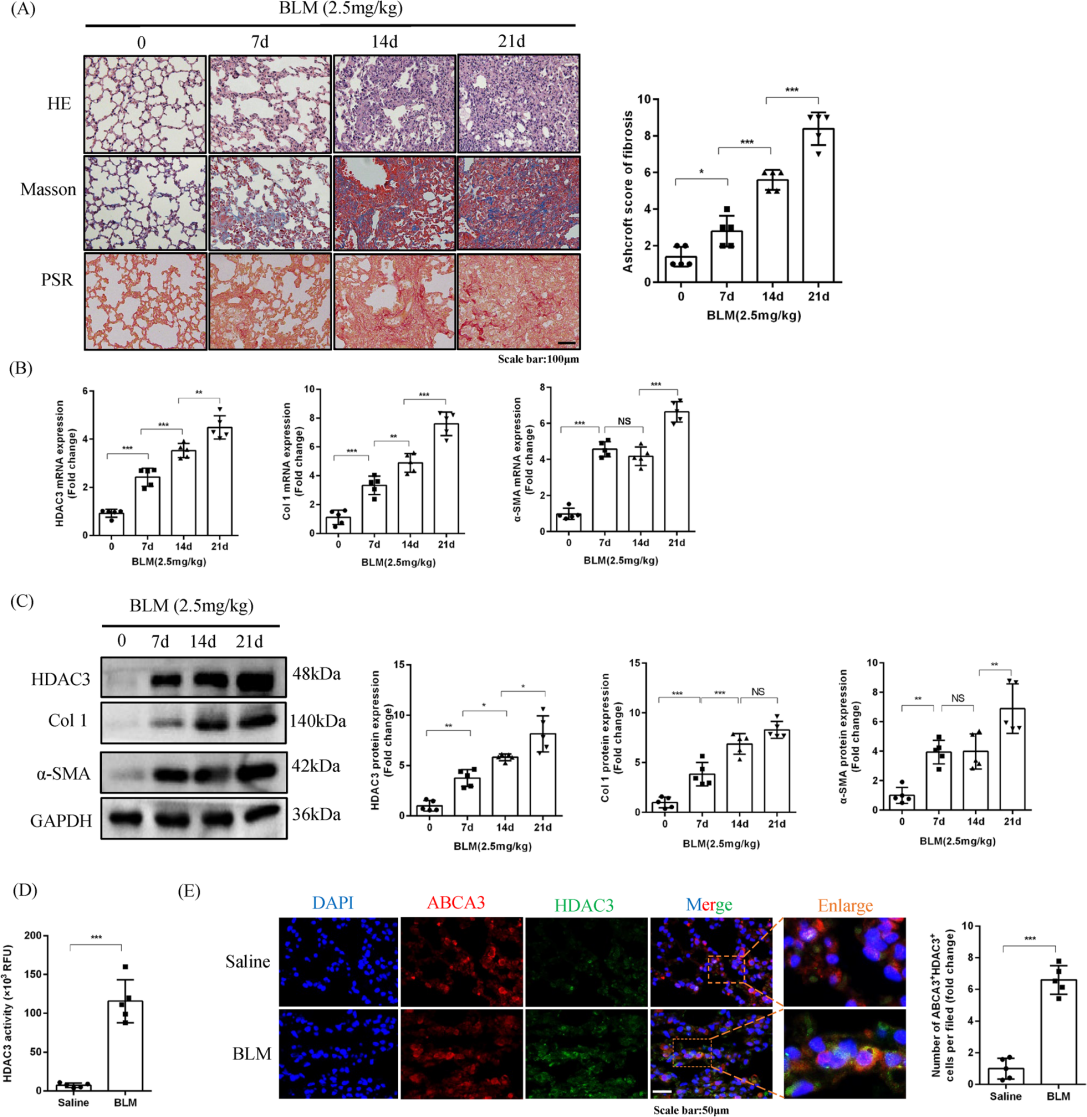
HDAC3 is upregulated in lung tissues and AT2 cells from mice with BLM-induced pulmonary fibrosis. **A** Histological analyses of H&E staining, masson staining, PSR staining and Ashcroft score for fibrosis in BLM-treated mice at day 0, 7, 14 and 21 post-treatment (× 200, n = 5). **B** Relative mRNA expression of HDAC3, Col 1 and α-SMA in lung tissues from BLM-treated mice at day 0, 7, 14 and 21 (n = 5). **C** Representative Western blot images and corresponding quantitative results for HDAC3, Col 1 and α-SMA in lung tissues from BLM-treated mice at day 0, 7, 14 and 21 (n = 5). **D** HDAC3 activity in lung tissues from saline and BLM-treated mice (2.5 mg/kg for 21d; n = 5). **E** Representative images showing HDAC3 and ABCA3 immunofluorescence colocalization staining and quantitation in lung tissues from BLM-treated mice at day 21 (× 400, n = 5). (*P < 0.05, **P < 0.01, ***P < 0.001, ^ns^*P* > 0.05)

Next, we further investigated the role of HDAC3 in pulmonary fibrosis in mice. As shown in Fig. 2A, pulmonary fibrosis following BLM stimulation gradually became more severe and peaked at day 21. The protein and mRNA expression levels of HDAC3, Col 1 and α-SMA were also increased in lung tissues from BLM-treated mice and peaked at day 21 (Fig. 2B, C). We also isolated the nucleus and cytoplasm of mouse lung tissue stimulated by BLM for 21 days, and found that HDAC3 protein expression increased in both the nucleus and cytoplasm (Additional file 1: Figure S1C). Meanwhile, HDAC3 activity was increased after BLM stimulation (Fig. 2D). Additionally, immunofluorescence results showed that HDAC3 was highly expressed in AT2 cells in lung tissues from mice with BLM-induced PF compared with the control group (Fig. 2E). These results further demonstrated that HDAC3 was involved in the pathogenesis of PF.

### TGF-β1/SMAD3 signaling upregulates HDAC3 transcription

**Fig. 3:**
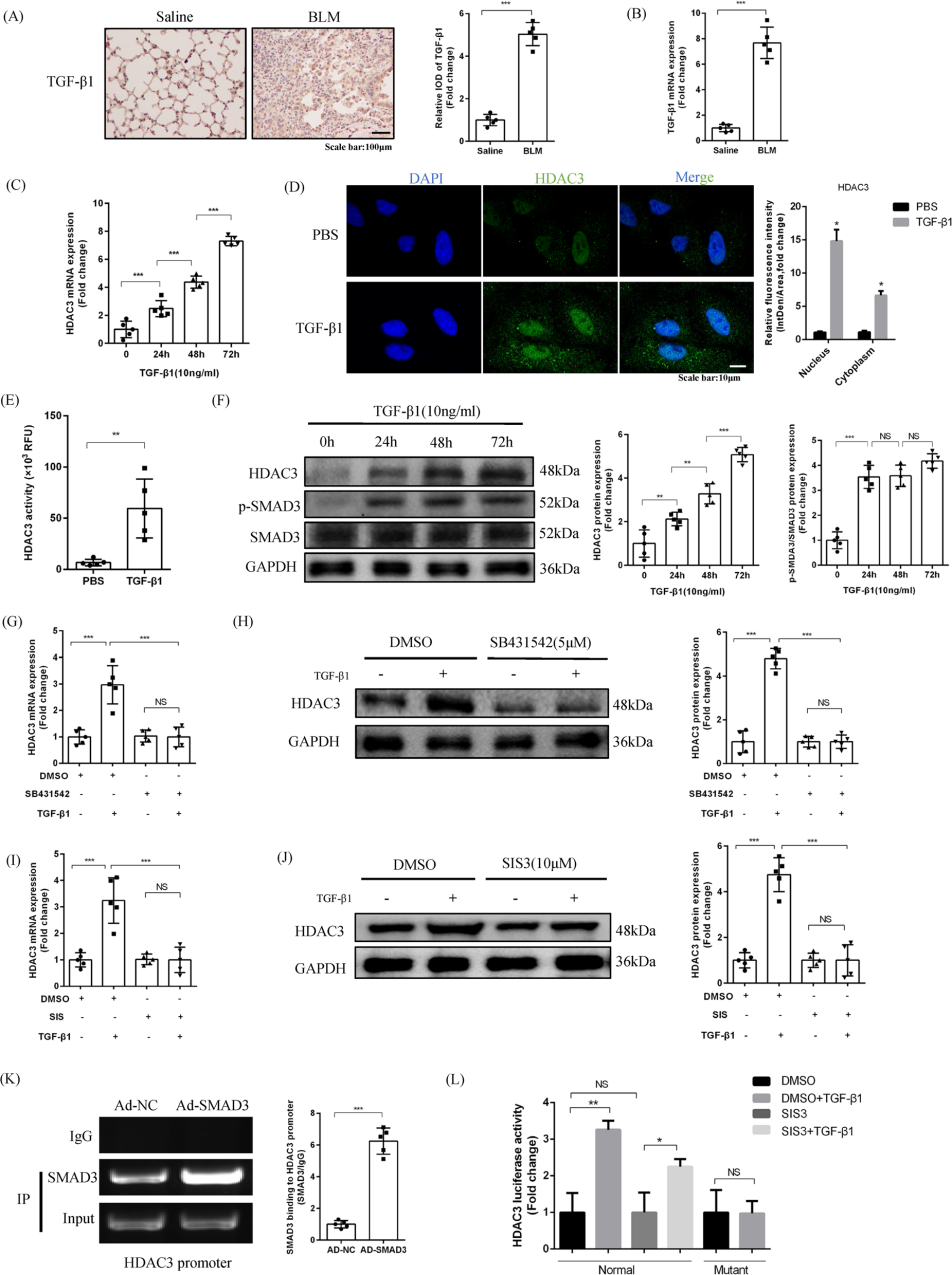
TGF-β1/SMAD3 signaling upregulates HDAC3 transcription. **A** Representative images showing immunohistochemical staining of TGF-β1 in saline-treated and BLM-treated mice at day 21. (× 200, n = 5). **B** The relative mRNA level of TGF-β1 in lung tissues from BLM-treated mice at day 21. (n = 5). **C** The relative mRNA level of HDAC3 in TGF-β1-treated primary mouse AT2 cells at 0, 24, 48, 72 h. (n = 5). **D** Representative images of HDAC3 immunofluorescence staining and quantitation in AT2 cells after TGF-β1 stimulation at 72 h. (× 1000, n = 5). **E** HDAC3 activity in AT2 cells after PBS or TGF-β1 treatment (10 ng/ml for 72 h). (n = 5). **F** Representative Western blot images and the corresponding quantitative results for HDAC3, phosphorylated-SMAD3 (p-SMAD3) and SMAD3 in TGF-β1-treated primary mouse AT2 cells at 0, 24, 48, 72 h. (n = 5). **G**, **H** mRNA and protein expression of HDAC3 after SB431542 inhibited TGF-βRI in AT2 in the presence of DMSO or TGF-β1 for 72 h. (n = 5) (SB431542 was administered half an hour before TGF-β1 or DMSO). **I**, **J** The mRNA and protein level of HDAC3 after SIS3 inhibited the phosphorylation of SMAD3 in AT2 in the presence of DMSO or TGF-β1 for 72 h. (n = 5; SIS3 was administered half an hour before TGF-β1 or DMSO). **K** Chromatin immunoprecipitation assay showing SMAD3 and the HDAC3 promoter with or without SMAD3 overexpression in AT2 cells. (n = 5). **L** Quantitation of HDAC3 via luciferase assay. AT2 cells were transfected with the normal murine HDAC3 promoter reporter plasmid (CAGCGAGCCCAGACATCTCGCTTGA) and mutant HDAC3 promoter reporter plasmid (CAGCGAGCCCAGCACTCTCGCTTGA) as a negative control plus a renilla luciferase plasmid. Then, AT2 cells were treated with TGF-β1 (10 ng/ml) and/or SIS3 (10 μM) for 72 h and luciferase activities measured and normalized with renilla luciferase activities(n = 5). (*P < 0.05, **P < 0.01, ***P < 0.001, ^ns^*P* > 0.05)

To investigate the possible cause of HDAC3 upregulation, we focused on the TGF-β1 signaling pathway since TGF-β1/SMAD3 signaling has previously been reported to directly promote HDAC3 transcription in renal fibrosis [17]. In vivo, immunohistochemistry showed that TGF-β1 was highly expressed in the lung tissues of mice with BLM-induced PF (Fig. 3A). TGF-β1 mRNA expression was also increased (Fig. 3B). In vitro, after TGF-β1 stimulation of primary mouse AT2 cells, HDAC3 mRNA expression steadily increased with time, reaching a peak at 72 h (Fig. 3C). Importantly, HDAC3 was increased in both the cytoplasm and nucleus after TGF-β1 stimulation (Additional file 1: Figure S1D). Immunofluorescence also showed increased HDAC3 expression (Fig. 3D) along with increased HDAC3 activity (Fig. 3E) in the presence of TGF-β1. Moreover, as time progressed following TGF-β1 treatment, HDAC3 protein expression gradually increased, accompanied by a high level of SMAD3 phosphorylation, both of which peaked at 72 h (Fig. 3F).

Next, we evaluated the activity of a selective inhibitor of TGF-β receptor I, SB431542. Treatment with SB431542 completely inhibited TGF-β1-induced upregulation of HDAC3 at the protein and mRNA levels (Fig. 3G, H). To further analyze downstream signals, we used SIS3, a selective inhibitor of SMAD3 phosphorylation, to investigate the relationship between TGF-β1/SMAD3 and HDAC3 signaling. The results showed that SIS3 completely inhibited the upregulation of HDAC3 protein and mRNA by TGF-β1 (Fig. 3I, J).

To analyze whether SMAD3 and the HDAC3 promoter directly combined, we conducted a ChiP-qPCR experiment. The results showed that overexpression of SMAD3 by adenovirus in AT2 cells could enhance the binding of SMAD3 to the HDAC3 promoter (Fig. 3K). Finally, we constructed a HDAC3 promoter reporter plasmid (HDAC3p-luc) and a mutant plasmid (mHDAC3p-luc) in which the SMAD3 binding motif CAGCGAGCCCAGACATCTCGCTTGA was replaced by CAGCGAGCCCAGCACTCTCGCTTGA. We found that TGF-β1 significantly induced the transactivation of HDAC3p-luc, but not the mutant mHDAC3p-luc. In addition, the TGF-β1-induced transactivation of HDAC3p-luc was blocked by SIS3 (Fig. 3L). These results indicated that TGF-β1 could directly up-regulate HDAC3 via SMAD3 signaling under fibrotic conditions.

### HDAC3 deficiency in AT2 cells alleviates pulmonary fibrosis in *vivo* and in *vitro* through suppression of EMT

**Fig. 4:**
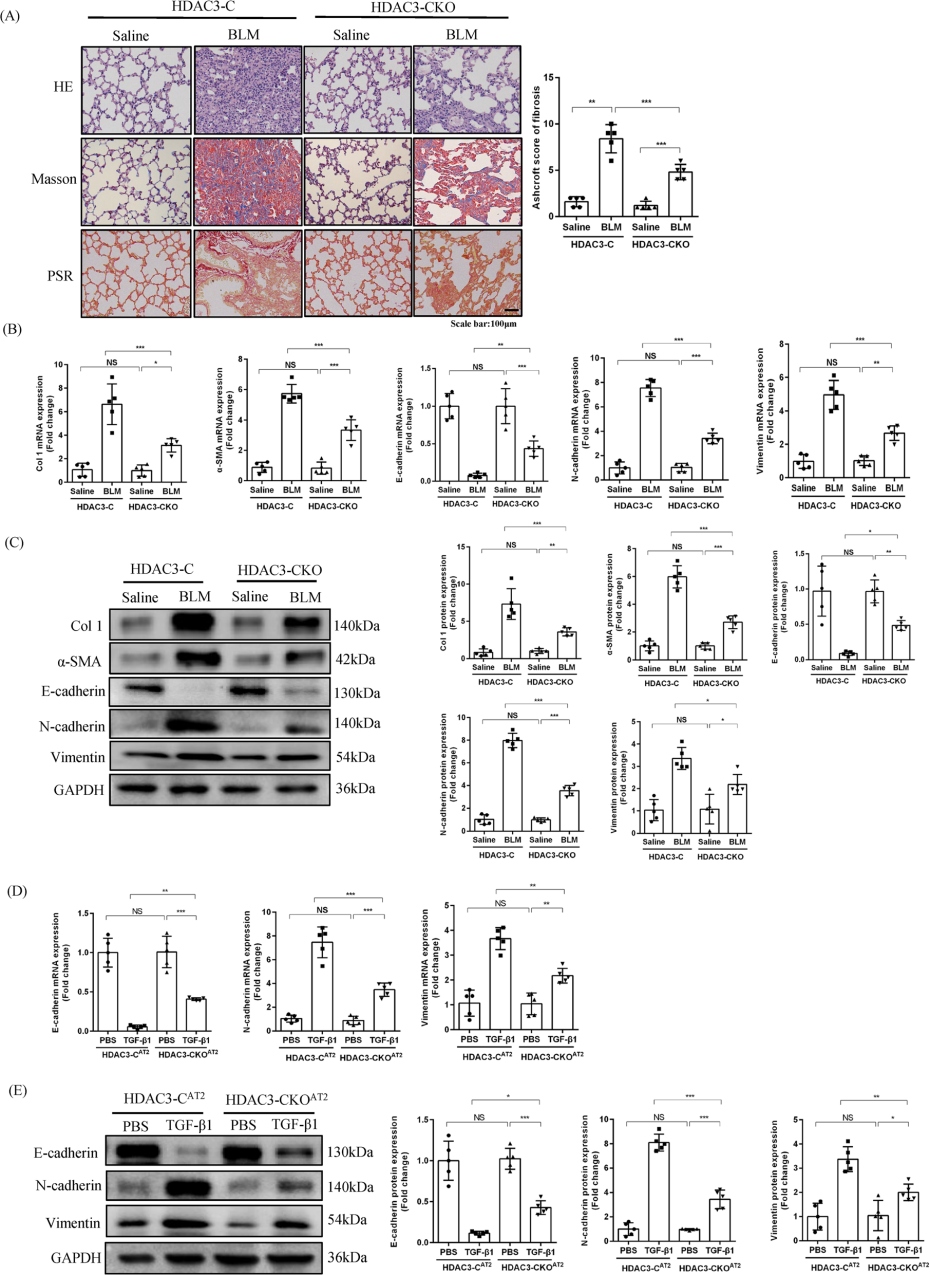
HDAC3 deficiency in AT2 cells alleviates pulmonary fibrosis in *vivo* and in *vitro* through suppression of EMT*.* **A** Histological analyses showing H&E staining, masson staining, PSR staining and Ashcroft score for fibrosis of BLM-treated mice at day 21 in HDAC3-C and HDAC3-CKO mice. (× 200, n = 5). **B** The relative mRNA expression of Col 1, α-SMA, E-cadherin, N-cadherin and vimentin in HDAC3-C and HDAC3-CKO mice in the presence of saline or BLM for 21d. (n = 5). **C** Representative Western blot images and the corresponding quantitative results for E-cadherin, N-cadherin and vimentin in HDAC3-C and HDAC3-CKO mice in the presence of saline or BLM for 21d. (n = 5). **D** The relative mRNA expression of E-cadherin, N-cadherin and vimentin in PBS-treated or TGF-β1 treated primary AT2 cells from HDAC3-C and HDAC3-CKO mice for 72 h. (n = 5). **E** Representative Western blot images and the corresponding quantitative results for E-cadherin, N-cadherin and vimentin in HDAC3-C and HDAC3-CKO mice in PBS-treated or TGF-β1 treated primary AT2 cells from HDAC3-C and HDAC3-CKO mice for 72 h. (n = 5). (*P < 0.05, **P < 0.01, ***P < 0.001, ^ns^*P* > 0.05)

To further confirm the potential roles of HDAC3 in mice with pulmonary fibrosis, we next generated AT2 cell-specific HDAC3-deficient mice by genetic engineering (Additional file 1: Figure S2A). HDAC3 was selectively deleted in AT2 cells, which was verified by PCR and agarose gel electrophoresis (Additional file 1: Figure S2B-C). Moreover, HDAC3 protein expression in lung tissues and primary AT2 cells from HDAC3-CKO mice as well as HDAC3-C mice was confirmed via Western blot to further verify the knockout effect (Additional file 1: Figure S2D-E). Under BLM stimulation, HDAC3-CKO mice showed significantly attenuated pulmonary fibrosis and decreased Ashcroft score for fibrosis, as illustrated by H&E staining, Masson’s trichrome staining, and PSR staining (Fig. 4A).

Next, to confirm the role of HDAC3 on EMT in pulmonary fibrosis, we evaluated the expression of fibrotic markers and EMT-related markers. Compared with HDAC3-C mice, HDAC3 deletion in AT2 cells reduced the expression of fibrotic markers (collagen 1 and α-SMA) and prevented EMT as evidenced by restoration of epithelial markers (E-cadherin) and depletion of mesenchymal markers (N-cadherin and vimentin) in vivo (Fig. 4B, C). Our in vitro experiments using primary AT2 cells from HDAC3-CKO mice (HDAC3-CKO^AT2^) and HDAC3-C mice (HDAC3-C^AT2^), stimulated by TGF-β1, showed that compared with HDAC3-C^AT2^, HDAC3 deficiency could prevent EMT as evidenced by restoration of epithelial markers (E-cadherin) and depletion of mesenchymal markers (N-cadherin and vimentin) (Fig. 4D, E). Collectively, these data suggested that HDAC3 deficiency in AT2 cells could inhibit BLM-induced pulmonary fibrosis by suppressing EMT.

### Genetic deletion or pharmacological inhibition of HDAC3 promotes the acetylation and degradation of GATA3

**Fig. 5:**
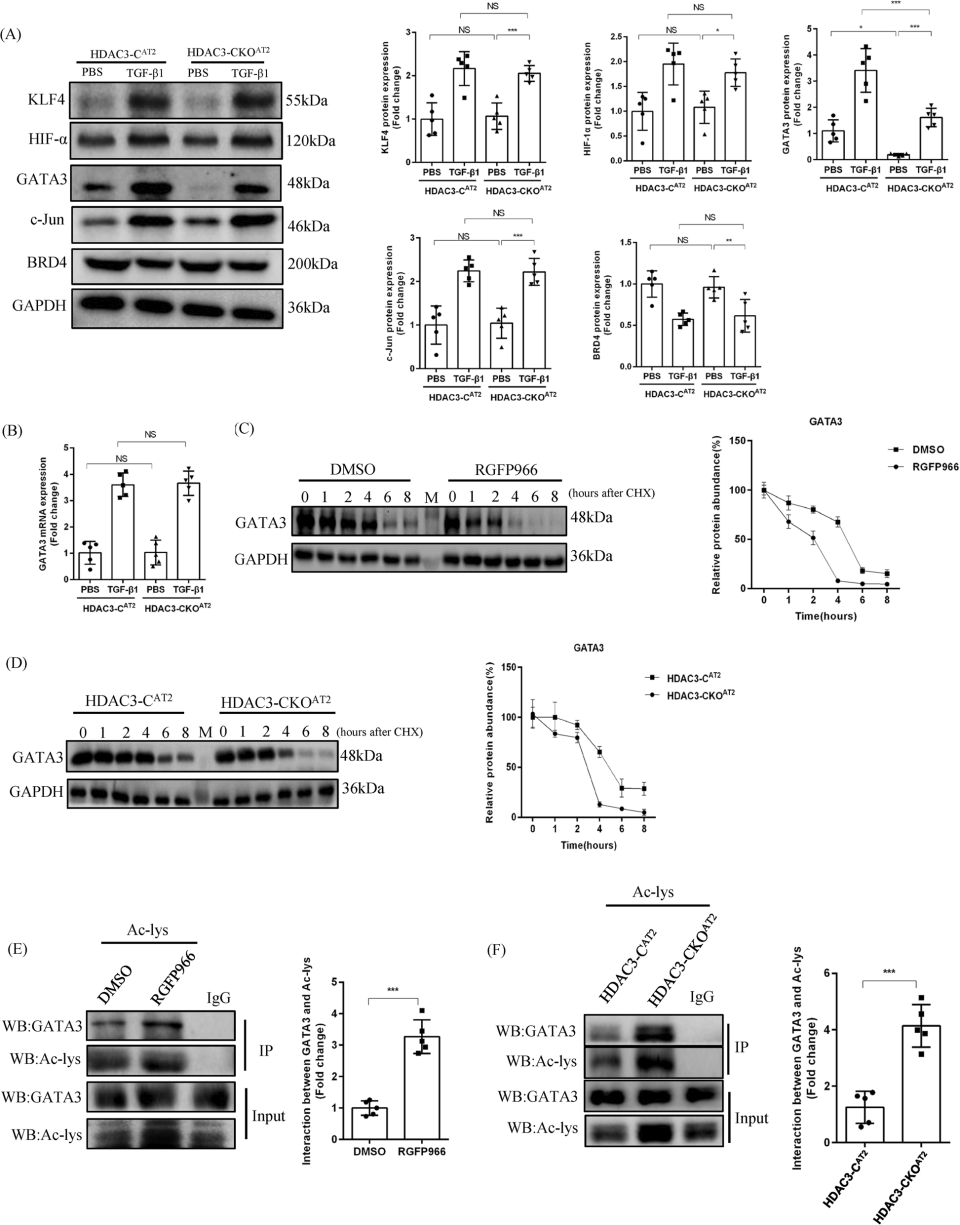
Genetic deletion or pharmacological inhibition of HDAC3 promotes the acetylation and degradation of GATA3. **A** Representative Western blot images and the corresponding quantitative results of KLF4, HIF-α, GATA3, c-Jun and BRD4 in PBS-treated or TGF-β1 treated primary AT2 cells from HDAC3-C and HDAC3-CKO mice for 72 h. (n = 5). **B** The relative mRNA expression of GATA3 in PBS-treated or TGF-β1 treated primary AT2 cells from HDAC3-C and HDAC3-CKO mice for 72 h. (n = 5). **C**, **D** Protein degradation assay. Representative Western blot images and corresponding quantitative results for GATA3 in AT2 cells following treatment with cycloheximide (250 μg/ml) for 0, 1, 2, 4, 6, 8 h after genetic (HDAC3 deficiency in AT2) or pharmacological (RGFP966, 15 μM) inhibition of HDAC3. (n = 3). **E** The acetylation level of GATA3 in AT2 was determined by Co-IP experiments after genetic (HDAC3 deficiency in AT2) or pharmacological (RGFP966 for 4 h, 15 μM) inhibition of HDAC3. (n = 5). (*P < 0.05, **P < 0.01, ***P < 0.001, ^ns^*P* > 0.05. M refers to protein marker)

Recent findings indicate that non-histones are frequently acetylated, resulting in changes in the subcellular localization, stability and enzymatic activity of non-histones as well as probable involvement in EMT [18]. To further clarify the mechanisms by which HDAC3 regulates EMT, we analyzed several non-histones such as KLF4, HIF-α, GATA3, c-Jun and BRD4, which when acetylated have been reported to directly impact EMT marker proteins [19]. Strikingly, as shown in Fig. 5A, the protein levels of KLF4, HIF-α, c-Jun, and BRD4 showed no significant changes in TGF-β1-stimulated AT2 cells from HDAC3-C^AT2^ mice compared with HDAC3-CKO^AT2^ mice. However, GATA3 was decreased in HDAC3-deficient AT2 cells in the presence of TGF-β1. Notably, GATA3 expression was reduced even in the absence of TGF-β1 in HDAC3-deficient AT2 cells. These data suggested that HDAC3 inhibition could reduce GATA3 protein expression under specific physiological and pathological conditions. To prove that HDAC3 affects only GATA3 protein expression and not mRNA, we first confirmed that HDAC3 had no impact on TGF-β1-induced changes in GATA3 mRNA levels (Fig. 5B). Next, we used cycloheximide, an inhibitor of protein synthesis in eukaryotes, to investigate the effect of HDAC3 on GATA3 protein levels. As shown in Fig. 5C, D, both genetic deletion and pharmacological inhibition of HDAC3 could accelerate the degradation of GATA3 protein. Next, to demonstrate that HDAC3 affects GATA3 protein expression by regulating its acetylation level, Co-IP analysis was performed. Co-IP showed that the acetylation of GATA3 was boosted both in the HDAC3-CKO^AT2^ and RGFP9660-treated groups (Fig. 5E, F). Taken together, these results suggested that HDAC3 may help to maintain the stability of GATA3 protein and prevent its degradation, thus preserving a low level of acetylation.

### TGF-β1/SMAD3/HDAC3 mediated EMT is partially dependent on GATA3

**Fig. 6:**
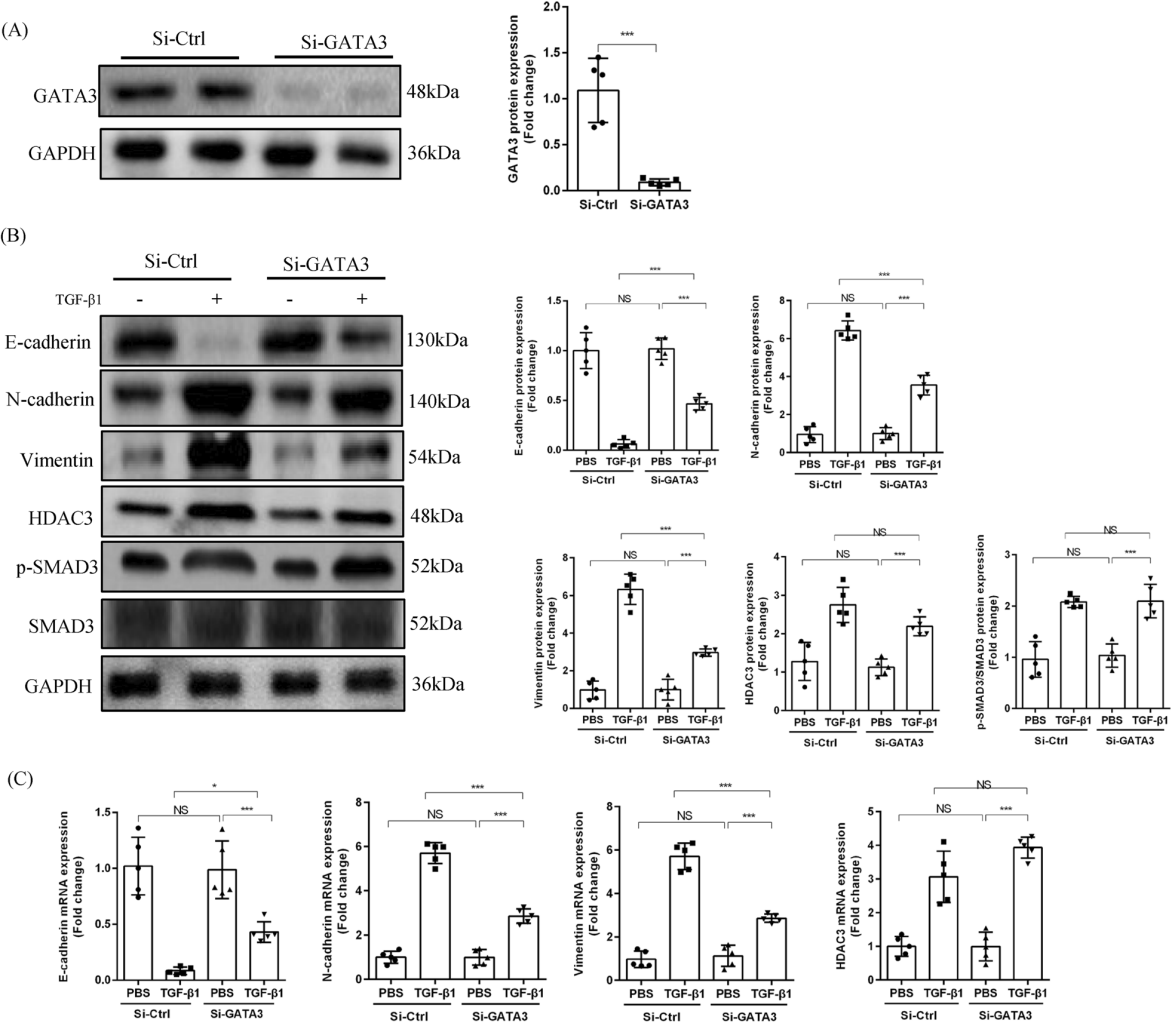
TGF-β1/SMAD3/HDAC3 mediated EMT is partially dependent on GATA3. **A** GATA3 protein quantification following knockdown by si-GATA3. (n = 5). **B** Representative Western blot images and the corresponding quantitative results of E-cadherin, N-cadherin, vimentin, HDAC3, p-SMAD3 and SMAD3 in PBS-treated or TGF-β1 treated primary AT2 cells for 72 h after GATA3 was knocked down or not. (n = 5). **C** Relative mRNA expression of HDAC3, E-cadherin, N-cadherin and vimentin. (n = 5). (*P < 0.05, **P < 0.01, ***P < 0.001, ^ns^*P* > 0.05)

To investigate whether TGF-β1/SMAD3/HDAC3-mediated EMT is completely dependent on GATA3, we successfully knocked down GATA3 in AT2 cells (Fig. 6A). As shown in Fig. 6B, C, GATA3 knockdown resulted in partial increases in E-cadherin protein and mRNA following TGF-β1 stimulation, without a return to baseline. Similarly, both N-cadherin and vimentin partially decreased and did not return to baseline levels. Additionally, both HDAC3 and phosphorylated SMAD3 protein and mRNA expression showed no significant changes after GATA3 knockdown, in the presence of TGF-β1. Collectively, these data demonstrated that TGF-β1/SMAD3/HDAC3 promoted EMT through GATA3, but that this process is not completely dependent on GATA3.

### The selective HDAC3 inhibitor, RGFP966, alleviated BLM-induced pulmonary fibrosis and EMT in vivo

**Fig. 7:**
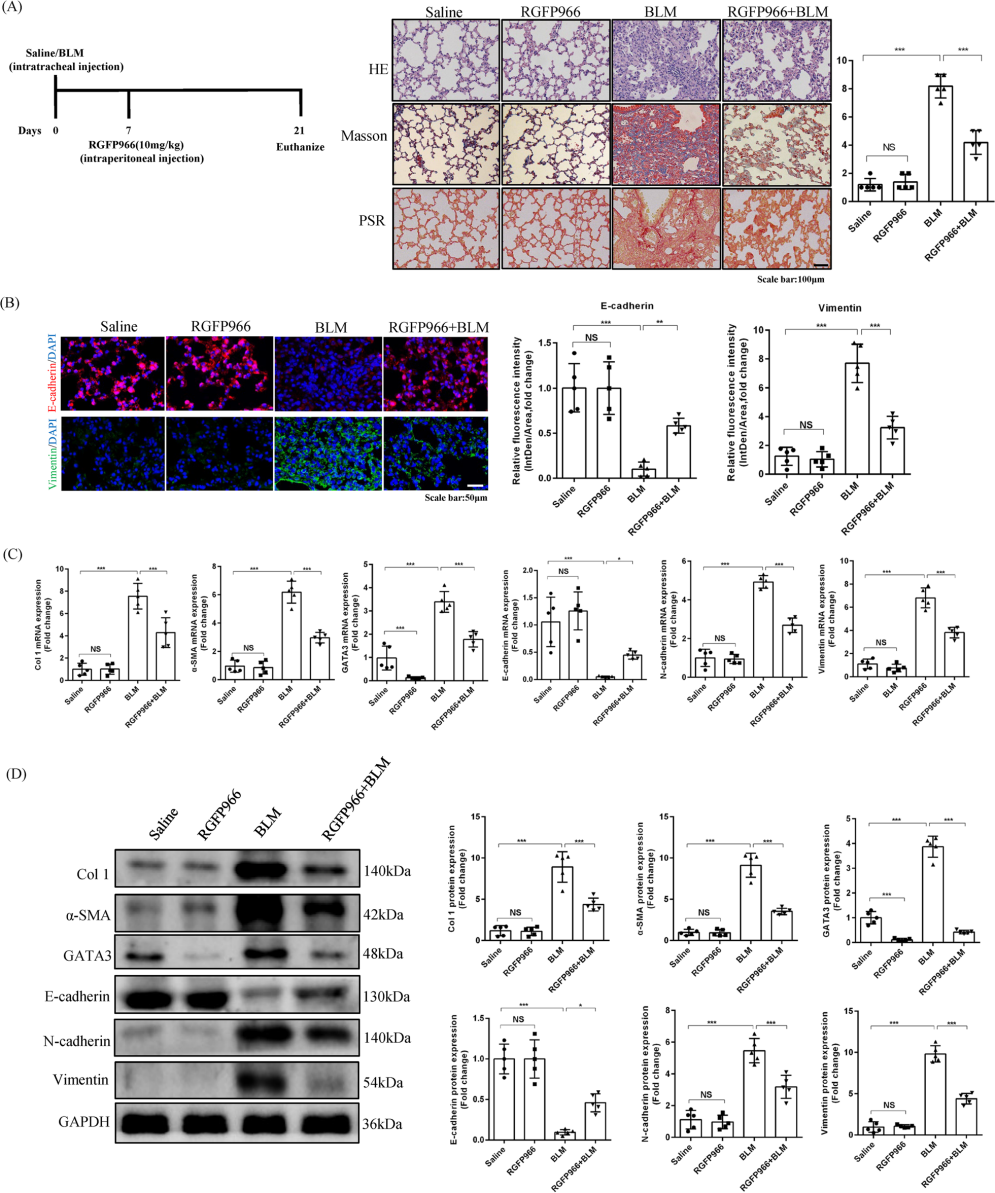
The selective HDAC3 inhibitor, RGFP966, alleviated BLM-induced pulmonary fibrosis and EMT in vivo*.* **A** Schematic diagram of RGFP966 intervention. Histological analyses using H&E staining, masson staining, PSR staining and Ashcroft score for fibrosis in saline-treated and BLM-treated mice at day 21 after RGFP966 was administered intraperitoneally. (× 200, n = 5). **B** Representative images of E-cadherin and vimentin immunofluorescence staining and quantitation in lung tissues from saline-treated and BLM-treated mice at day 21 after RGFP966 was administered intraperitoneally. (× 400, n = 5). **C**, **D** The relative mRNA and protein level of Col 1, α-SMA, E-cadherin, N-cadherin, vimentin and GATA3 in lung tissues from saline-treated and BLM-treated mice at day 21 after RGFP966 was administered intraperitoneally. (n = 5). (*P < 0.05, **P < 0.01, ***P < 0.001, ^ns^*P* > 0.05)

Finally, we explored the potential translation of our findings into a therapeutic strategy against pulmonary fibrosis. As shown in Fig. 7A, intraperitoneal injection of RGFP966 on day 7 after BLM stimulation significantly alleviated pulmonary fibrosis in mice, as evidenced by H&E staining, Masson’s trichrome staining and PSR staining. Immunofluorescence also confirmed the inhibition of EMT, showing upregulation of E-cadherin expression and downregulation of vimentin expression (Fig. 7B). Furthermore, at the protein and mRNA levels, we demonstrated that RGFP966 reduced the expression of collagen 1 and α-SMA, and prevented EMT as evidenced by restoration of E-cadherin and depletion of N-cadherin and vimentin. Notably, RGFP966 was still able to promote GATA3 protein degradation in the absence of BLM, which is consistent with our previous in vitro results. RGFP966 also significantly reduced GATA3 protein expression in the presence of BLM (Fig. 7C, D). Similarly, Co-IP results showed that RGFP966 increased the acetylation level of GATA3 in BLM-treated mice (Additional file 1: Figure S3A). To demonstrate that RGFP966 mainly targets HDAC3, we detected the protein expression of HDAC3, HDAC2, HDAC4, and SIRT3, respectively. The results showed that in the context of fibrosis, RGFP966 significantly reduced the expression of HDAC3, but had no significant effect on the expression of HDAC2, HDAC4, and SIRT3 (Additional file 1: Figure S3B). Overall, these data showed that administration of the selective HDAC3 inhibitor RGFP966 may serve as a promising therapeutic strategy against pulmonary fibrosis in clinical practice.

## Discussion

**Fig. 8:**
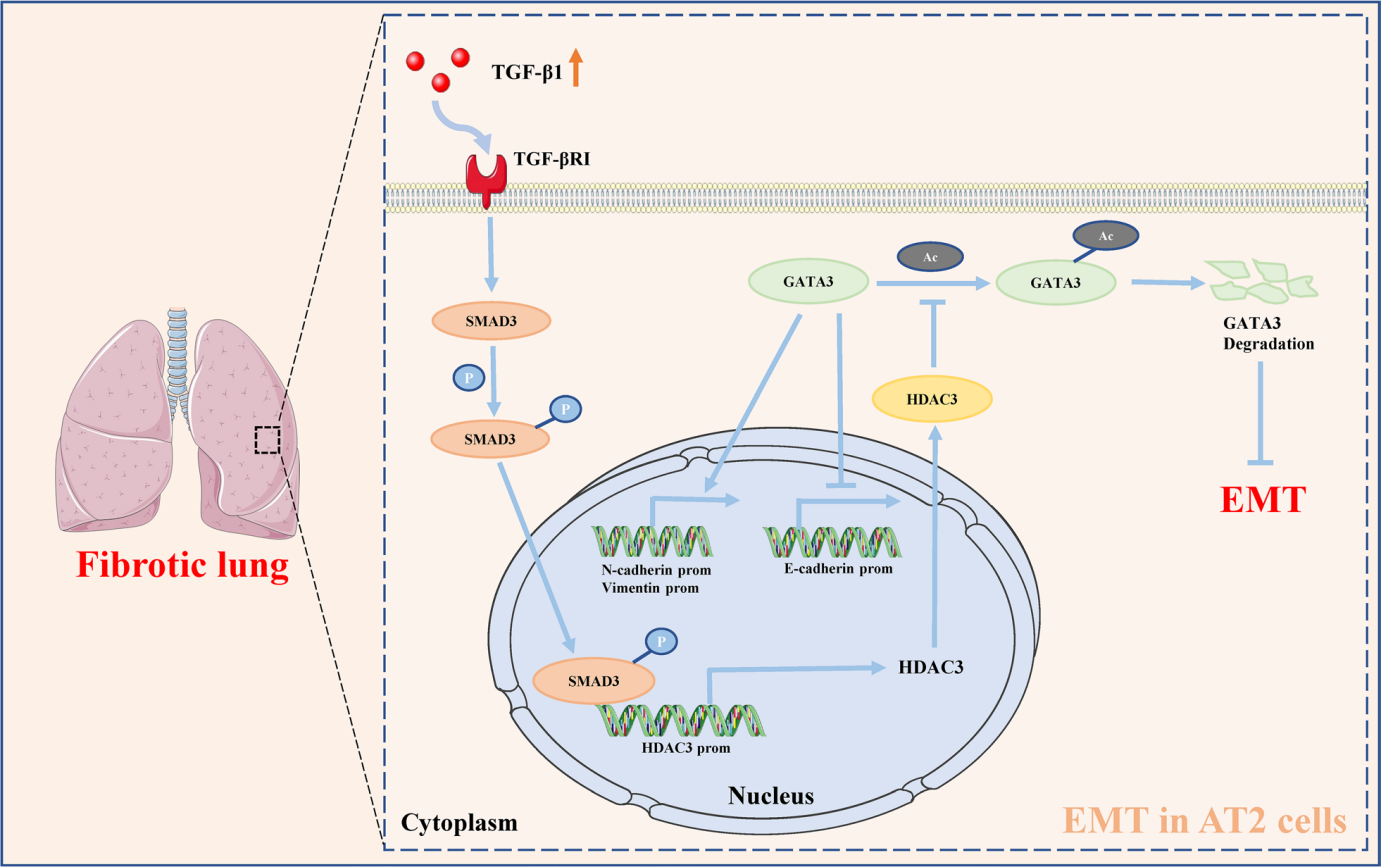
Schematic illustration of the mechanisms of HDAC3 in AT2 cells. TGF-β1 expression is upregulated in pulmonary fibrosis and phosphorylates SMAD3 through the TGF-βRI receptor. Activated SMAD3 directly binds to the promoter of HDAC3 to increase its transcription. HDAC3 further decreases the acetylation of GATA3 and inhibits its degradation, which promotes the epithelial-mesenchymal transition of AT2 cells by decreasing E-cadherin expression and increasing expression of N-cadherin and vimentin, which aggravates pulmonary fibrosis

In this study, we confirmed high expression of HDAC3 in lung tissues from patients with IPF, as well as mice with BLM-induced pulmonary fibrosis, and cellular fibrosis models. Most importantly, HDAC3 was highly expressed in AT2 cells. Moreover, AT2-specific deletion of HDAC3 or intraperitoneal injection of the selective HDAC3 inhibitor, RGFP966, significantly alleviated EMT in mice with BLM-induced pulmonary fibrosis. Consistent with in vivo data, HDAC3 inhibition in primary mouse AT2 cells exerted similar protective effects on TGF-β1-induced EMT. In terms of mechanisms, we showed that TGF-β1/SMAD3 directly promoted HDAC3 transcription. HDAC3 acts on GATA3, maintaining a low level of acetylation, preserving its stability, and protecting against degradation, thus enhancing EMT (Fig. 8). Meanwhile, inhibition of HDAC3 results in increased GATA3 acetylation, which reduces its stability and promotes its degradation, resulting in attenuated EMT. Based on these data, we propose that HDAC3 may be a potential therapeutic target for the prevention of pulmonary fibrosis.

PF poses a huge threat to public health. Some patients develop PF due to identifiable triggers such as silicosis, pneumoconiosis, and aging [20, 21], but other cases of PF have unknown origins, and are consequently termed “idiopathic” (IPF). Studies have shown that both alveolar macrophages and AT2 cells are effector cells of pulmonary fibrogenesis. Wang et al. [22] found that MBD2 is a viable target for PF treatment through inhibition of the macrophage M2 program. Further, Sisson et al. [23] found that targeted injury of AT2 cells induces PF. Based on these previous studies, we considered whether HDAC3 is highly expressed and functional in M2 macrophages in pulmonary fibrosis. Immunofluorescence showed that the expression of HDAC3 in the M2 macrophages from patients with IPF was not significantly different compared with normal controls (Additional file 1: Figure S4A). Collectively, these data indicate that HDAC3 is highly expressed and plays a role in AT2 cells in pulmonary fibrosis.

There is a consensus in the literature that the TGF-β1/SMAD3 signaling pathway plays a pivotal role in the pathogenesis of IPF [24, 25]. TGF-β1 is up-regulated and activated in fibrotic diseases and triggers a pro-fibrotic response via activation of the Smad-2/3 cascade [26]. Our study adds to this body of evidence by demonstrating the upregulation of HDAC3 by TGF-β1 both in vivo and in vitro. We found that TGF-β1 could phosphorylate SMAD3 through TGF-β receptor I, and that activated SMAD3 could directly bind to the HDAC3 promoter to increase its transcription.

EMT, a process by which epithelial cells undergo a phenotypic conversion that leads to myofibroblast formation, plays a crucial role in the progression of IPF [14]. Moreover, AT2 cells undergoing EMT are the main source of myofibroblasts [7]. Therefore, given the high expression of HDAC3 in AT2 cells in the context of fibrosis in humans, mice, and primary mouse cells, we hypothesized that HDAC3 plays a role in EMT. We verified that HDAC3 deficiency in AT2 cells alleviated BLM-induced pulmonary fibrosis in mice and TGF-β1-induced EMT in vitro, as evidenced by increased E-cadherin expression and decreased N-cadherin and vimentin expression.

In view of the pivotal role of HDAC3 in regulating EMT in pulmonary fibrosis, we investigated the possible mechanism by which HDAC3 regulates EMT. Several possible transcription factors such as KLF4, HIF-α, GATA3, c-Jun, and BRD4 were screened, as the acetylation and deacetylation of these proteins has previously been reported to directly impact EMT marker proteins, such as E-cadherin and vimentin [19]. However, in our study, only GATA3 was regulated by HDAC3. Specifically, in both fibrosis and normal physiology, the protein expression of GATA3 decreases after HDAC3 inhibition. HDAC3 itself is known to deacetylate both histones and non-histones [18], whereas histone deacetylation mainly controls gene transcription and non-histone deacetylation regulates enzyme activity, protein stability and subcellular localization [27]. Next, to evaluate whether that HDAC3 regulates GATA3 expression at the protein but not mRNA level, RT-PCR was performed. We found that the mRNA level of GATA3 was not affected by HDAC3, in both the presence and absence of TGF-β1. This was corroborated by a protein degradation assay, which further illustrated that genetic deletion or pharmacological inhibition of HDAC3 accelerated the degradation of GATA3. Crucially, Co-IP experiments confirmed that the acetylation level of GATA3 increased following inhibition of HDAC3 through genetic or pharmacological means. Taken together, these data show that HDAC3 promotes EMT through GATA3, mainly by maintaining low-level acetylation of GATA3 to preserve its protein stability and prevent degradation. Consequently, the expression of E-cadherin is reduced and the expression of N-cadherin and vimentin is increased, thus promoting EMT. However, our experiments also showed that the induction of EMT by HDAC3 was not completely dependent on GATA3, as the protein levels of E-cadherin, N-cadherin, and vimentin, did not return to baseline levels after GATA3 was knocked down in TGF-β1-induced AT2 cells.

It is possible that HDAC3 can also regulate EMT through other pathways. For example, Jeong et al. found that HDAC3 may accelerate pulmonary fibrosis progression by enhancing EMT in alveolar cells through the regulation of miR-224 and FOXA1 [14]. Specifically, HDAC3 induces miR-224 expression to drive alveolar EMT through FOXA1 downregulation. In addition, Ma et al. found that down-regulated HDAC3 elevates microRNA-495-3p to restrain EMT and the oncogenicity of melanoma cells via reduction of TRAF5 expression [28]. Of course, whether this mechanism contributes to pulmonary fibrosis remains to be verified. Another study showed that under hypoxic conditions, HDAC3 interacts with hypoxia-induced WDR5, recruits the histone methyltransferase (HMT) complex to increase histone H3 lysine 4 (H3K4)-specific HMT activity, and activates mesenchymal gene expression [29], which is likely to contribute to pulmonary fibrosis.

Notably, some studies have suggested that reprogramming of AT2 cells is required to restore AT1 cells in IPF [30, 31], and that transient EMT is involved in that reprogramming process. In light of this, it would be pertinent to understand if/how inhibiting HDAC3 (which blocked EMT in AT2 cells) impacts the restoration of AT1 cells in BLM-induced mice.

Next, we examined several AT1 markers such as AQP5, AGER and HOPX by qPCR in HDAC3-C and HDAC3-CKO mice in the presence of saline or BLM. Interestingly, as shown in Additional file 1: Figure S5, these AT1 markers decreased after HDAC3 knockdown in mice with BLM-induced PF. Based on our results, we suggest that after deletion of HDAC3 in AT2 cells, EMT is inhibited, and AE1 reconstruction is damaged, which facilitates fibrosis. However, it is the inhibition of EMT that reduces more fibroblast foci and thus reduces pulmonary fibrosis, which is a protective effect, and this protective effect is greater than the adverse effect. Therefore, we propose that HDAC3 deletion in AT2 cells may play a role in reducing pulmonary fibrosis.

Finally, to understand the potential clinical application of our research, we performed a simple drug experiment. RGFP966, a selective inhibitor of HDAC3, was injected intraperitoneally on day 7 after BLM stimulation. Encouragingly, RGFP966 significantly alleviated BLM-induced pulmonary fibrosis in mice and impaired EMT progression in AT2 cells. Notably, treatment with RGFP966 is not specific for AT2 cells and previous research has identified over-expression of HDAC3 in pulmonary fibroblasts from patients with IPF [32]. Thus, we isolated positive and negative EpCAM-cells from lung tissues of BLM-treated mice at day 21. In positive EpCAM-cells, RGFP966 significantly reduced the expression of N-cadherin and vimentin, and increased the expression of E-cadherin. At the same time, the expression of the fibrotic markers, Col1 and α-SMA, was significantly reduced (Additional file 1: Figure S6A). In negative EpCAM-cells, RGFP966 significantly reduced the expression of fibrotic markers Col1 and α-SMA, and the expression of mesenchymal markers N-cadherin and vimentin, but had no effect on the expression of the epithelial marker E-cadherin (Additional file 1: Figure S6B). To further validate our findings, we performed a double-IF-staining for HDAC3 and vimentin in bleomycin-injured lungs (Additional file 1: Figure S6C). The results showed that HDAC3 was significantly increased in fibroblasts in the context of fibrosis. More importantly, our bioinformatics analysis and immunofluorescence experiments identified the increased expression of HDAC3 in pulmonary fibroblasts from patients with IPF compared with healthy controls (Additional file 1: Figure S7). Taken together, our data indicate that in addition to increased HDAC3 in AT2 cells contributing to pulmonary fibrosis, the increased HDAC3 in fibroblasts may also play an important role in the pathogenesis of pulmonary fibrosis. RGFP966 may also alleviate pulmonary fibrosis by inhibiting HDAC3 expression in fibroblasts.

## Conclusion

This study demonstrated that the activation of the TGF-β1/SMAD3 signaling pathway was directly responsible for the up-regulation of HDAC3 expression in pulmonary fibrosis. More importantly, HDAC3 played a pivotal role in mediating EMT in AT2 cells and pulmonary fibrosis by deacetylating GATA3 and inhibiting its degradation. Therefore, targeted inhibition of HDAC3 in AT2 cells may provide a new therapeutic opportunity for the prevention of pulmonary fibrosis.

## Materials and methods

### Reagents and antibodies

Bleomycin hydrochloride (HY-17565A), (E)-SIS3(HY-13013), SB-431542 (HY-10431), RGFP966 (HY-13909) and cycloheximide (HY-12320) were purchased from MedChemExpress (Shanghai, China). Antibodies against HDAC3, Collagen 1, TGF-β1, α-SMA, E-cadherin, N-cadherin, Vimentin, KLF4, HIF-1α, GATA3, c-Jun, BRD4, pan acetyl-lysine, IgG, ABCA3, CD206, HDAC2, HDAC4, SIRT3, EpCAM and GAPDH were provided by ABclonal (Wuhan, China). Antibodies against SMAD3 and phosphorylated (P)-SMAD3 were purchased from Cell Signaling Technology (Danvers, MA, USA). Secondary antibodies against HRP-conjugated AffInipure Goat Anti-Rabbit IgG (H + L), HRP-conjugated AffInipure Goat Anti-Mouse IgG (H + L) Cy3-conjugated AffInipure Goat Anti-Rabbit IgG (H + L) and FITC conjugated AffInipure Goat Anti-Rabbit IgG (H + L) were purchased from Proteintech Group, Inc (Wuhan, China). HDAC3 plasmid, SMAD3 mimic and negative control were obtained from GenePharma Co.,Ltd (Shanghai, China). QuickMutation™Plus Site-Directed Mutagenesis Kit, Dual-Lumi™Luciferase Reporter Gene Assay Kit and Immunoprecipitation Kit with Protein A + G Agarose Gel were provided by Beyotime Biotechnology (Shanghai, China). SMAD3 adenovirus was purchased from Hanheng Biotechnology (Shanghai) Co., Ltd. Other general chemical reagents were purchased from Sinopharm Chemical Reagent Co., Ltd (Shanghai, China).

### Human samples

We collected lung tissue samples from six male patients who were diagnosed with IPF and underwent lung transplantation at Renmin Hospital of Wuhan University. Lung tissues around the pulmonary bulla from six male patients < 20 years old were collected as healthy controls. A part of each fresh lung tissue sample was fixed with paraformaldehyde for histological analysis, and the remaining part was frozen at − 80 ℃ for molecular biology analysis. This study was approved by the Clinical Research Ethics Committee of Renmin Hospital of Wuhan University (WDRY2022-K207). All subjects gave informed consent.

### Animals and treatment

All animal experiments were approved by the Laboratory Animal Welfare & Ethics Committee of Renmin Hospital of Wuhan University (20221205A). All the mice were kept in the Animal Center of Renmin Hospital of Wuhan University in a specific pathogen-free (SPF) barrier system with a humidity of 45–55% and a temperature of 20–25 °C on a regular 12 h light/dark cycle. All animal experiments were conducted at the Animal Center of Renmin Hospital of Wuhan University. C57BL/6 mice were obtained from the Hubei Provincial Center for Disease Control and Prevention. (Wuhan, China). HDAC3^flox/−^ mice were purchased from Shanghai Model Organisms Center, inc. (Shanghai, China). Sftpc-CreERT2( ±) and transgenic mice were purchased from GemPharmatech Co., Ltd (Jiangsu, China). To generate AT2 cell-specific HDAC3-deficient (HDAC3-CKO) mice, Sftpc-CreERT2( ±) mice were crossed with HDAC3^flox/−^ mice. Littermate HDAC3^flox/flox^Sftpc-CreERT2(-/-)(HDAC3-C) mice were used as controls (Additional file 1: Figure S2).

Tamoxifen treatment was administered to induce the deletion of HDAC3 in AT2 cells, HDAC3-CKO mice were administered intraperitoneal (IP) injections of 100 mg/kg/day tamoxifen (TAM, Sigma-Aldrich) diluted in absolute ethanol and corn oil (Sigma-Aldrich; v/v 1:9) for 5 days. The HDAC3-C mice were treated the same way. To establish a pulmonary fibrosis model in *vivo*, the mice were given intratracheal injection of bleomycin(2.5 mg/kg) dissolved in 50 μl sterile saline as previously reported [33]. The control group was administered the same volume of sterile saline via the trachea. Of note, for the HDAC3-CKO mice, bleomycin was administered immediately after the last tamoxifen-treatment. For RGFP966 treatment, RGFP966 (10 mg/kg body weight) was administered by intraperitoneal injection after BLM stimulation [15]. The mice were euthanized on days 0, 7, 14 and 21. Specifically, the mice were sacrificed by cervical dislocation at the end of the experiment under deep anesthesia with 0.3% pentobarbital sodium. The right lung was quickly taken out and frozen at − 80 ℃ for molecular biological analysis. The intact left lung was dissected and perfused with paraformaldehyde through the trachea for histological analysis.

### Isolation and culture of EpCAM-positive and negative cells

Briefly, after euthanasia, the neonatal mice were soaked in 75% ethanol and disinfected for 5 min. In a sterile state, cut open the chest cavity layer by layer, cut off the entire lung, and wash the blood on the surface of the lung tissue with sterile PBS solution. Then cut the lung tissue into 1 mm^3^ sized tissue blocks and place them in a 10 ml centrifuge tube. Add 6 ml of 0.2% collagenase and digest at 37 °C for 1 h. Then add 4 ml of 0.5% trypsin and digest for 30 min. Centrifuge at 4 °C for 5 min (1500r/min) and discard the supernatant. Add 5 ml of Ham's F-12 K culture medium containing 10% FBS to terminate digestion, blow evenly, filter the cell suspension with a 74 μm well metal filter, and adjust the cell concentration to (2 ~ 3) × 10^6^ cells/ml. Pre coat EpCAM antibody in cell culture dishes. Plant the cell suspension in the culture dish and incubate it at 37 °C for 6 h. The cells adsorbed in the dish are EpCAM-positive cells, and the supernatant is EpCAM-negative cells.

The extracted EpCAM-positive cells were cultured at 37℃ and 5% CO_2_, with 10% FBS + Ham’s F-12 K; EpCAM-negative cells were cultured at 37℃ and 5% CO_2_, with 10% FBS + PRMI-1640.

### Isolation and culture of AT2 cells

Neonatal mouse primary AT2 cells were isolated by membrane filtration and immune-adhesion [34]. Firstly, after euthanasia, the mice were disinfected with alcohol and the lung tissues were removed and digested with trypsin and collagenase, and red blood cell lysis buffer was added to remove red blood cells. Next, preliminary isolation was performed according to the size of the cells. AT2 cells were about 10 μm smaller than macrophages (about 25 μm) and AT1 cells (50-100 μm). Large tissue debris was removed by primary filtration through a 74 μm membrane. All AT1 cells and some macrophages were removed by subsequent filtration through a 38 μm membrane. Finally, a filter membrane with a pore size of 19 μm was used for further fine filtration. The filtrate was purified by immune-adhesion. A plastic plate was coated with IgG, and the cell suspension was placed on the plate. This technique causes macrophages and most other non-AT2 cells to bind to the IgG due to their Fc fragment-containing receptors, and become adsorbed to the plate. In contrast, non-adherent AT2 cells are washed down to obtain AT2 cells with high purity. AT2 cells extracted from HDAC3^flox/flox^ mice were labeled as HDAC3-C^AT2^, and AT2 cells extracted from HDAC3-CKO mice were labeled as HDAC3-CKO^AT2^.

The extracted AT2 cells were cultured at 37℃ and 5% CO_2_, with 10% FBS + Ham’s F-12 K for 72 h, during which the growth state and cell morphology were quantified (Additional file 1: Figure S8). The HDAC3 selective inhibitor RGFP966 (15 μM), TGF-β1 (10 ng/ml), inhibitor of TGF-β receptor type I (TGF-βRI) SB-431542 (5 μM) and inhibitor of SMAD3 phosphorylation SIS3 (10 μM) were added at the appropriate time.

### Bioinformatics analysis of HDAC3 expression in human AT2 cells

The GSE86618 dataset, which comprises single-cell transcriptomic datasets of AT2 cells isolated from healthy individuals or patients with IPF, was analyzed [35].In addition, the GSE180415 dataset, which comprises RNA sequencing datasets of lung fibroblasts isolated from healthy individuals or patients with IPF, was also analyzed. The preprocessed table with normalized expression measurements was downloaded using the GEOquery package [36] within the R statistical programming environment. Differences between groups were analyzed for statistical significance by non-parametric Wilcoxon signed-rank test and were visualized as scatter plots.

### Histopathological analysis

The left lung was fixed with paraformaldehyde, paraffin embedded and sectioned, dewaxed and hydrated, and then dehydrated and sealed after being stained with hematoxylin and eosin (H&E).

Next, Masson’s trichrome staining was performed as follows: the sections were dewaxed and incubated in methyl dichromate overnight, and then stained with iron hematoxylin, ponceau red acid fuchsin, phosphomolybdic acid and aniline blue in turn. Finally, the sections were dehydrated and sealed for microscopic examination.

Picrosirius red (PSR) staining was also conducted. Briefly, sections were placed in xylene I for 20 min. followed by xylene II for 20 min, absolute ethanol I for 5 min, absolute ethanol II for 5 min, and finally 75% alcohol for 5 min. After washing with tap water, the sections were stained in Sirius red dye for 8 min, dehydrated and sealed, and observed under the microscope.

The severity of interstitial fibrosis was assessed in a blinded fashion by two researchers using the Ashcroft scoring system [37].

### Immunohistochemistry

Immunohistochemical analysis was performed as previously described [38]. Briefly, 3 μm-thick paraffin sections of mouse lung were prepared, followed by dewaxing, hydration, blocking, antigen retrieval, and further blocking with 5% BSA for 30 min at room temperature. Then, slides were incubated overnight with an appropriate concentration of primary antibody at 4 ℃. After washing with PBS three times, the secondary antibody was added and incubated at 37 °C for 60 min. After washing again with PBS, DAB was added for 5–10 min, and samples were rinsed with tap water for 5 min. The expression of corresponding protein in lung tissue was observed and captured by microscopy. ImageJ software was used to quantify protein abundance.

### Immunofluorescence

ABCA3 is a specific marker of AT2 cells [39]. First, 3-μm paraffin sections of lung tissue were prepared and subjected to dewaxing, hydration, antigen retrieval, and washing with PBS, before incubating in primary antibody (anti-ABCA3, 1:50, purchased from Abcam in Cambridge, #ab32369; anti-HDAC3, 1:50, purchased from Abcam in Cambridge, #ab99856) overnight at 4 °C. The next day, slides were rewarmed and washed again with PBS, and then incubated in secondary antibody for 60 min at room temperature in the dark. Slides were then washed again in PBS and DAPI was added to stain the nuclei, incubating for 30 min at room temperature in the dark. Slides were washed again in PBS and then finally sealed with gum medium for observation under the confocal microscope (Olympus, Japan).

AT2 cells were fixed with 4% paraformaldehyde for 20 min, washed with PBS, and blocked with 5% goat serum for 30 min at room temperature. Then, cells were permeabilized using 0.5% Triton X-100 for 20 min and washed with PBS, and incubated in primary antibody (anti-HDAC3,1:50) overnight at 4 ℃. The next day, samples were rewarmed, washed with PBS, then incubated in an appropriate amount of secondary antibody for 60 min at room temperature in the dark. After washing again with PBS, DAPI was added to stain the nuclei, followed by washing in PBS again and sealing with neutral gum. Samples were observed and images captured using a confocal microscope (Olympus, Japan).

#### Real-time PCR

Real-time PCR was performed as previously described [40]. After extracting total RNA, reverse transcription was conducted with the following reaction mix: total RNA 2 µl, 5 × Reaction buffer 4 µl, SweScript RT I Enzyme Mix 1 µl, Oligo (dT)_18_ Primer (100 μM) 1 µl, and DEPC water added to a total volume of 20 µl. The reaction conditions were: 50 ℃ for 20 min and 85 ℃ for 5 s. The PCR reaction system included: SYBR Green Realtime PR Master Mix 10 µl, forward and reverse primers 0.8 µl, cDNA template 2 µl, and ddH_2_O added to a total volume of 20 µl, The PCR reaction conditions were: initial denaturation at 95 °C for 30 s, then 40 cycles of (95 °C for 5 s, 60 °C for 10 s, 72 °C for 15 s). The cycle threshold (CT) value was determined and the 2^−△△CT^ method was used to calculate the relative expression of mRNA. PCR primer sequences are shown in Table 1.

**Table 1:** The primers used in real-time PCR

Species	Gene	Forward primer	Reverse primer
Mice	*HDAC3*	ATGGCATTGATGACCAGAGTTACA	CAACATTTCGGACAGTGTAGCC
Mice	*Collagen 1*	AAGAAGCACGTCTGGTTTGGAG	GGTCCATGTAGGCTACGCTGTT
Mice	*α-SMA*	GTACCACCATGTACCCAGGC	GAAGGTAGACAGCGAAGCCA
Mice	*CDH1*	CGACCGGAAGTGACTCGAAAT	TCAGAACCACTGCCCTCGTAAT
Mice	*CDH2*	CCCTGACTGAGGAGCCTATGAA	GGTTGATAATGAAGATGCCCGTT
Mice	*Vimentin*	GCAGTATGAAAGCGTGGCTG	CTCCAGGGACTCGTTAGTGC
Mice	*TGF-β1*	TAATGGTGGACCGCAACAAC	CCACATGTTGCTCCACACTTGAT
Mice	*GATA3*	GGAAGAGGTGGACGTACTTTTTAAC	AGAGATCCGTGCAGCAGAGG
Mice	*AGER*	TCCCGATGGCAAAGAAACACT	GCAGGAGAAGGTAGGATGGGT
Mice	*AQP5*	CACATCAATCCGGCCATTACTC	CGCATTGACGGCCAGGTTA
Mice	*HOPX*	AGGTGGAGATCCTGGAGTACA	AAGGCAAGCCTTCTGACCG
Mice	*GAPDH*	CCTCGTCCCGTAGACAAAATG	TGAGGTCAATGAAGGGGTCGT
Human	*HDAC3*	TGGTGGTTATACTGTCCGAAATG	ATCTGGTCCAGATACTGGCGTG
Human	*GAPDH*	GGAAGCTTGTCATCAATGGAAATC	TGATGACCCTTTTGGCTCCC

#### Western blot

Western blotting was performed as previously described [41]. Briefly, murine lung tissue or AT2 cells from the experimental interventions described above were treated with lysis buffer, placed in an automatic homogenizer on ice for 30 min, and fully lysed. After centrifugation at 4 ℃, the supernatant was collected and the protein concentration was measured with a BCA assay kit. Loading buffer was added to the protein samples, which were then boiled to denature the proteins. Samples were immediately cooled, then resolved by SDS-PAGE electrophoresis. After transfer, the membrane was turned, rinsed and blocked. The membrane was then incubated overnight at 4 °C in the appropriate concentration of primary antibody (HDAC3-1:1000, Collagen 3–1:1000, α-SMA-1:1000, E-cadherin-1:1000, N-cadherin:1:1000, Vimentin-1:1000, SMAD3-1:1000, p-SMAD3-1:1000, KLF4-1:1000, HIF-1α-1:1000, GATA3-1:1000, c-Jun-1:1000, BRD4-1:1000, GAPDH-1:5000). The next day, after rinsing, membranes were incubated in secondary antibody for 1.5 h. Membranes were rinsed again and images obtained by chemiluminescence. The gray values of protein expression were analyzed using Image J software as previously described [42].

#### Nuclear and cytoplasmic protein separation

Nuclear and cytoplasmic proteins were extracted with a nuclear and cytoplasmic protein extraction kit (P0028) (Beyotime Biotechnology, Shanghai, China) according to the manufacturer’s protocol. Proteins were analyzed by western blot.

#### Coimmunoprecipitation (Co-IP) analysis

Co-IP was performed using an immunoprecipitation kit and anti-pan acetyl-lysine (Ac-lys) antibody following the manufacturer’s protocol. Target proteins (GATA3 and Ac-lys) in the immunoprecipitants were examined by western blot. Co-IP of IgG was used as a negative control.

#### Protein degradation assay

To examine the degradation of GATA3 proteins using a pharmacological approach, AT2 cells pre-treated with DMSO or RGFP966 were incubated in cycloheximide (250 μg/ml) for 0, 1, 2, 4, 6, or 8 h and analyzed by western blot for GATA3 expression. For the genetic approach, HDAC3-C^AT2^ and HDAC3-CKO^AT2^ were also treated with cycloheximide (250 μg/ml) for 0, 1, 2, 4, 6, or 8 h and analyzed by western blot for GATA3 expression.

#### siRNA transfection

AT2 cells cultured in Ham’s F-12 K supplemented with 10% FBS were transfected with GATA3 siRNA or negative control siRNA (Sangon, Shanghai, China) using Lipofectamine 3000 (Invitrogen, Carlsbad, CA, USA) as previously described [43]. The transfected cells were then stimulated with murine TGF-β1 (10 ng/ml).

#### Chromatin immunoprecipitation (ChiP) assays

The base binding sites of SMAD3 and HDAC3 promoter were obtained through the starBase database. The putative SMAD3 binding motif is CAGCGAGCCCAGACATCTCGCTTGA. To verify this hypothesis, SMAD3 was upregulated via Ad-SMAD3 transfection in AT2 cells. Thirty-six hours after transfection, the cells were collected and cross-linked with paraformaldehyde (4%) for 10 min (37℃). Glycine (2 mg/mL) was added to neutralize additional paraformaldehyde followed by cell lysis using an ultrasonic apparatus. Antibodies against SMAD3 and IgG were used for immunoprecipitation. Immunoprecipitated DNA was used as a template for real time quantitative PCR reactions. At the same time, the amplified DNA was subjected to agarose gel electrophoresis to detect binding of SMAD3 to the HDAC3 promoter.

#### Dual-luciferase reporter gene assay

Mouse HDAC3 promoter reporter plasmid (HDAC3p-luc) was constructed in pGL-4-luc plasmid by inserting a PCR-amplified mouse genomic DNA fragment at XhoI and HindIII sites. Then, mutant plasmid (mHDAC3p-luc) in which the SMAD3 responsive element CAGCGAGCCCAGACATCTCGCTTGA was mutated to CAGCGAGCCCAGCACTCTCGCTTGA was generated by QuickMutation™Plus Site-Directed Mutagenesis Kit. Specifically, targeting plasmid template (CAGCGAGCCCAGACATCTCGCTTGA), the mutation primer (forward primer: CAGCGAGCCCAGCACTCTCGCTTGA; reverse primer: GTCGCTCGGGTCGTGAGAGCGAACT) was designed. Next, the primer was configured to 100 μm. Then, the gene site-specific mutation reaction was carried out, and the reaction system proceeded as follows: 10 × BeyoAmp™ Buffer (with Mg^2+^) 5 ul, forward primer and reverse primer 1 μl each, dNTP mix 5 μl, mutated plasmid 2 μl, BeyoAmp™ Extra-long DNA Polymerase 1 μl, Nuclease-Free Water 35 μl to a total volume of 50 μl. The PCR reaction procedure was as follows: 95℃, 3 min; 95℃, 30 s; 55℃, 30 s; 68℃, 30 s/kb; repeat the above 3 steps for 20 cycles; 68℃, 10 min; 4℃, hold on. Finally add DpnI for digestion. Next, 1 μg HDAC3p-luc / mHDAC3p-luc plasmid and 50 nmol/L SMAD3 mimic/NC plasmid were transfected into 1 × 10^4^ mouse AT2 cells. After 72 h, luciferase activity was calculated using the Renilla luciferase system.

#### HDAC3 activity assay

For human lung tissue and mouse lung tissue, the supernatant was collected after homogenization and centrifugation. For AT2 cells, RIPA lysate was added and centrifuged to extract the supernatant. HDAC3 activity was examined with a HDAC3 activity assay kit (Sigma, EPI004) according to the manufacturer’s instructions. Briefly, HDAC3 deacetylates the substrate [R-H–K-K (Ac) − AFC] to release AFC, which is fluorometrically detected (Ex/Em = 380/500 nm).

#### Statistical analysis

All the data in this study were expressed as the mean ± standard error of the mean and analyzed using SPSS 23.0 software. Unpaired Student’s t-test was used for comparisons between two groups. Two-way analysis of variance (ANOVA) without repeated measures was performed for multiple comparisons with two independent variables. One-way ANOVA with Tukey’s post hoc test was used for comparisons among three or more groups. Tukey's tests were run only when F achieved *P* < 0.05 and homogeneity of variance was satisfied; otherwise, Tamhane’s T2 post hoc test was performed. Normality and homogeneity were tested using Liljefors’ and Levene’s tests, respectively. Statistical significance was set at *P* < 0.05.

### Supplementary Information

**Additional file 1. Fig S1.** Distribution of HDAC3 in the nucleus and cytoplasm. **Fig S2.** Knockout effect of HDAC3 in mouse AT2 cells. **Fig S3.** Under the background of bleomycin-induced PF in mice. (A) The effect of RGFP966 on the acetylation of GTAT3 (B) and the expression of HDAC3, HDAC2, HDAC4, and SIRT3 proteins. **Fig S4.** Expression of HDAC3 in M2 macrophages in IPF. **Fig S5.** The relative mRNA expression of AQP5, AGER, and HOPX in HDAC3-C and HDAC3-CKO mice in the presence of saline or bleomycin for 21d. **Fig S6.** (A–B) Expression of fibrotic markers and EMT markers in positive and negative EpCAM-isolated cells respectively (C) HDAC3 and vimentin immunofluorescence colocalization staining in bleomycin-induced PF in mice. **Fig S7.** Bioinformatics and immunofluorescence staining analysis of HDAC3 expression in fibroblasts from patients with IPF vs healthy controls. **Fig S8.** Culture and identification of primary mouse AT2 cells.

## Data Availability

All data that support the findings in this study are available from the corresponding author upon reasonable request.
