# Supplementary material for: Histone deacetylase 3 deletion in alveolar type 2 epithelial cells prevents bleomycin-induced pulmonary fibrosis
Source: Clin Epigenetics. 2023 Nov 11;15:182. doi: 10.1186/s13148-023-01588-5 (PMC10640740; doi:10.1186/s13148-023-01588-5)
Supplement: Supplementary file 1 — Additional file 1. Fig S1. Distribution of HDAC3 in the nucleus and cytoplasm. Fig S2. Knockout effect of HDAC3 in mouse AT2 cells. Fig S3. Under the background of bleomycin-induced PF in mice. (A) The effect of RGFP966 on the acetylation of GTAT3 (B) and the expression of HDAC3, HDAC2, HDAC4, and SIRT3 proteins. Fig S4. Expression of HDAC3 in M2 macrophages in IPF. Fig S5. The relative mRNA expression of AQP5, AGER, and HOPX in HDAC3-C and HDAC3-CKO mice in the presence of saline or bleomycin for 21d. Fig S6. (A–B) Expression of fibrotic markers and EMT markers in positive and negative EpCAM-isolated cells respectively (C) HDAC3 and vimentin immunofluorescence colocalization staining in bleomycin-induced PF in mice. Fig S7. Bioinformatics and immunofluorescence staining analysis of HDAC3 expression in fibroblasts from patients with IPF vs healthy controls. Fig S8. Culture and identification of primary mouse AT2 cells. [file 13148_2023_1588_MOESM1_ESM.docx]

**Supplementary materials**


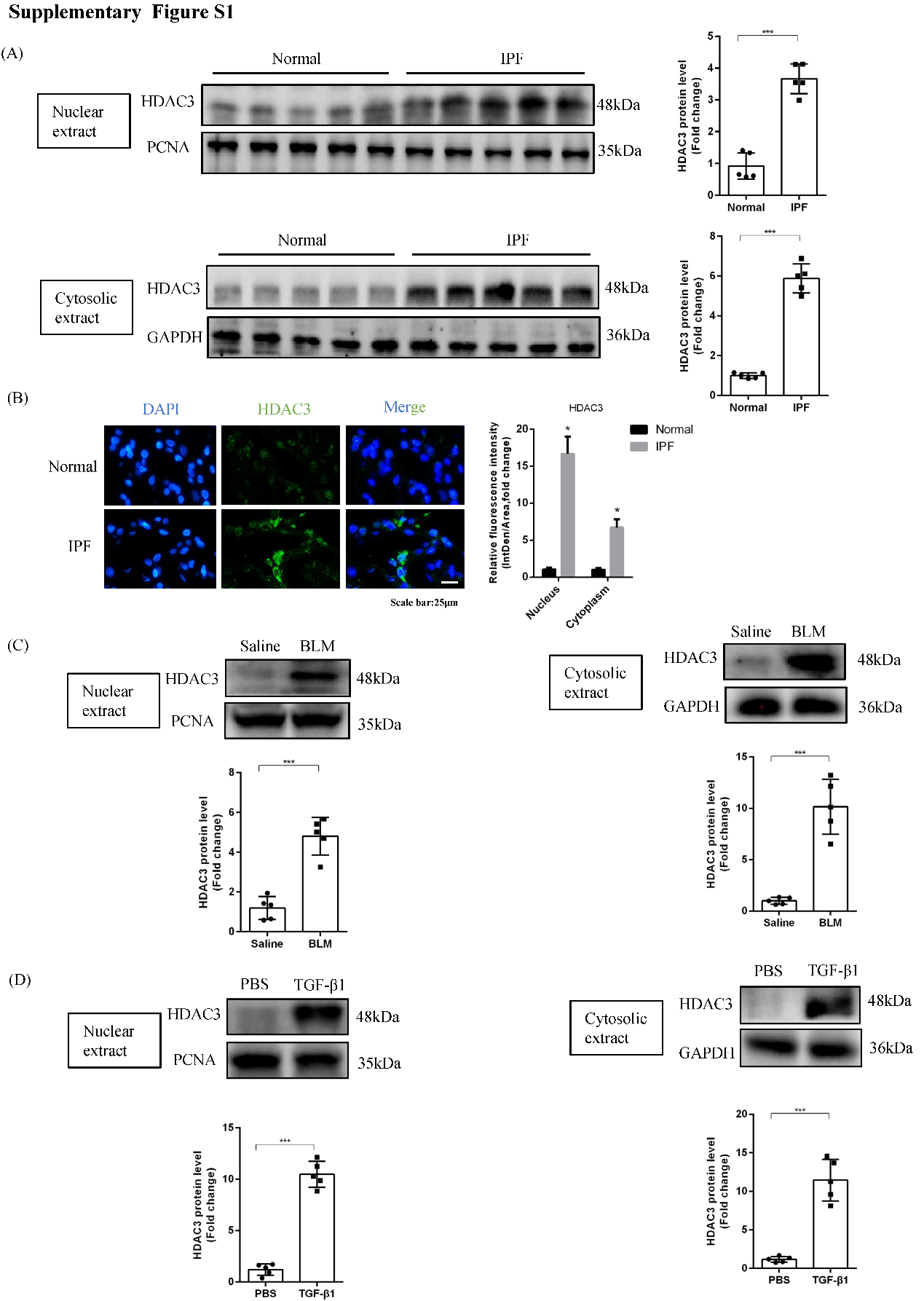


**Figure S1 (A).** The representative western blot images and the corresponding quantitative results of HDAC3 in cytosolic extract and nuclear extract of lung tissues from healthy controls and IPF patients. (n=5). **(B)** The representative confocal microscopy images show the relationship between HDAC3 and the nucleus. This image is from the enlarged portion of Figure 1F. (n=6, **P* < 0.05 versus Normal group) **(C).** The representative western blot images and the corresponding quantitative results of HDAC3 in cytosolic extract and nuclear extract of lung tissues from saline and BLM-treated mice (2.5mg/kg for 21d). (n=5). **(D)** The representative western blot images and the corresponding quantitative results of HDAC3 in cytosolic extract and nuclear extract of AT2 cells after PBS and TGF-β1 treated (10ng/ml for 72h) (n=5). (**P* < 0.05, ***P* < 0.01, ****P* < 0.001).


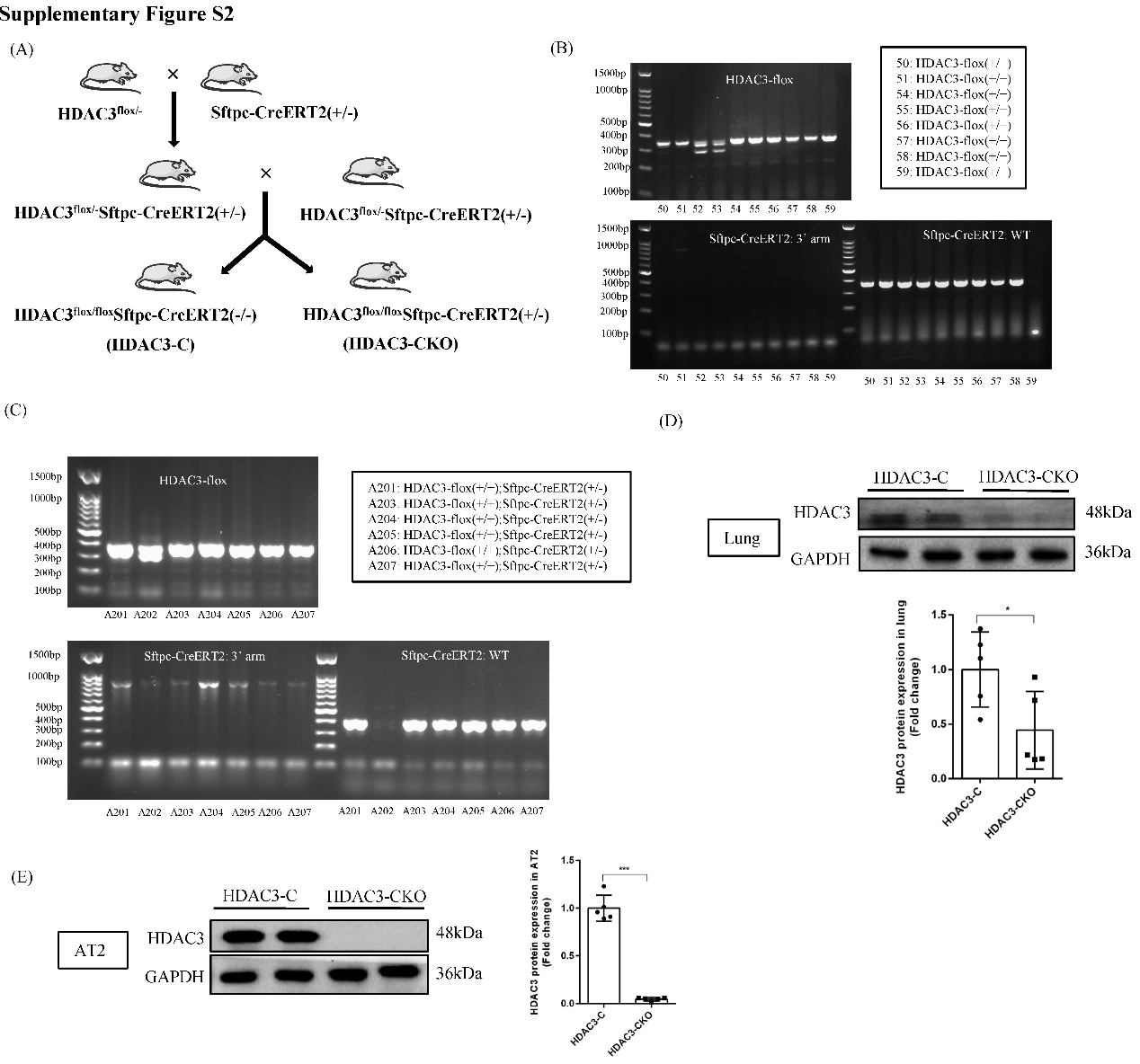


**Figure S2. (A).** HDAC3^flox/-^ mice crossed with Sftpc-CreERT2 (+/-) mice to generate HDAC3^flox/-^ Sftpc-CreERT2 (+/-) mice. Then, HDAC3^flox/-^ Sftpc-CreERT2 (+/-) mice crossed with HDAC3^flox/-^ Sftpc-CreERT2 (+/-) to generate the AT2-specific HDAC3-knockout mice, named as HDAC3^flox/flox^Sftpc-CreERT2 (+/-) (HDAC3-CKO). And HDAC3^flox/flox^ mice served as control mice (HDAC3-C)**.(B).** Agarose gel electrophoresis showed that mice numbered 50,51,54,55,56,57,58,59 were HDAC3^flox/flox^ (HDAC3-C) mice. (n=8). **(C)**. Agarose gel electrophoresis showed that mice numbered A201, A203, A204, A205, A206, A207 were HDAC3^flox/flox^ Sftpc-CreERT2 (+/-) (HDAC3-CKO) mice. (n=6). **(D-E).** Western blot analysis of HDAC3 in lung tissues and primary AT2 cells from HDAC3-C and HDAC3-CKO mice to verify the knock-out effect of HDAC3 in AT2 cells. HDAC3 is slightly expressed in the lung tissue of HDAC3-CKO mice, and almost not expressed in the AT2 cells of HDAC3-CKO mice. (n=5). (**P* < 0.05, ***P* < 0.01, ****P* < 0.001).


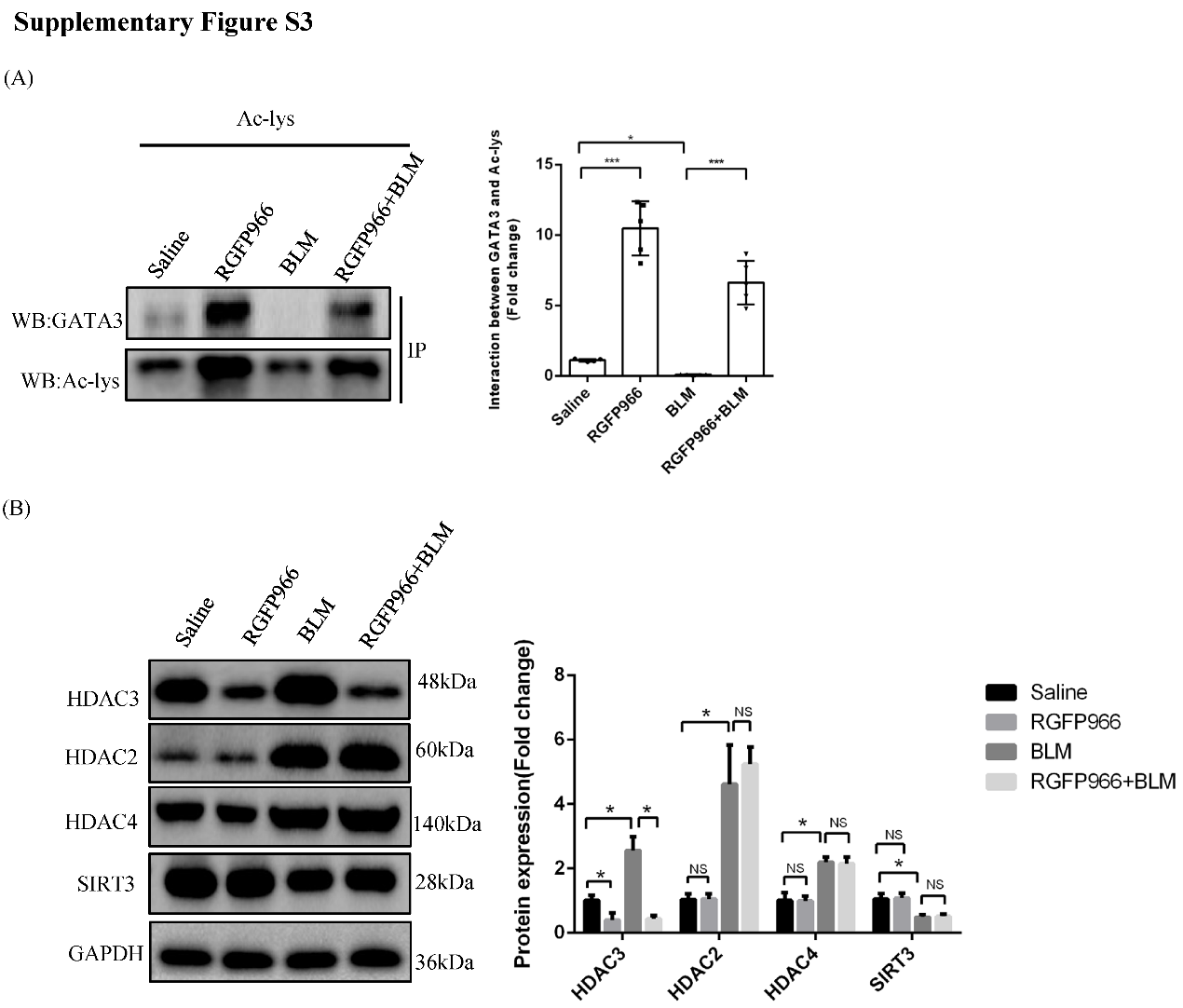


**Figure S3. (A).** The acetylation level of GATA3 in lung tissues was determined by Co-IP experiments after saline-treated and BLM-treated mice at day 21 after RGFP966 was administered intraperitoneally. (n=5). **(B).** The representative western blot images and the corresponding quantitative results of HDAC3, HDAC2, HDAC4 and SIRT3 in each group. (n=5). (Lung tissues derived from saline-treated and BLM-treated mice at day 21 after RGFP966 was administered intravenously) (**P* < 0.05, ***P* < 0.01, ****P* < 0.001, ^ns^*P*>0.05).


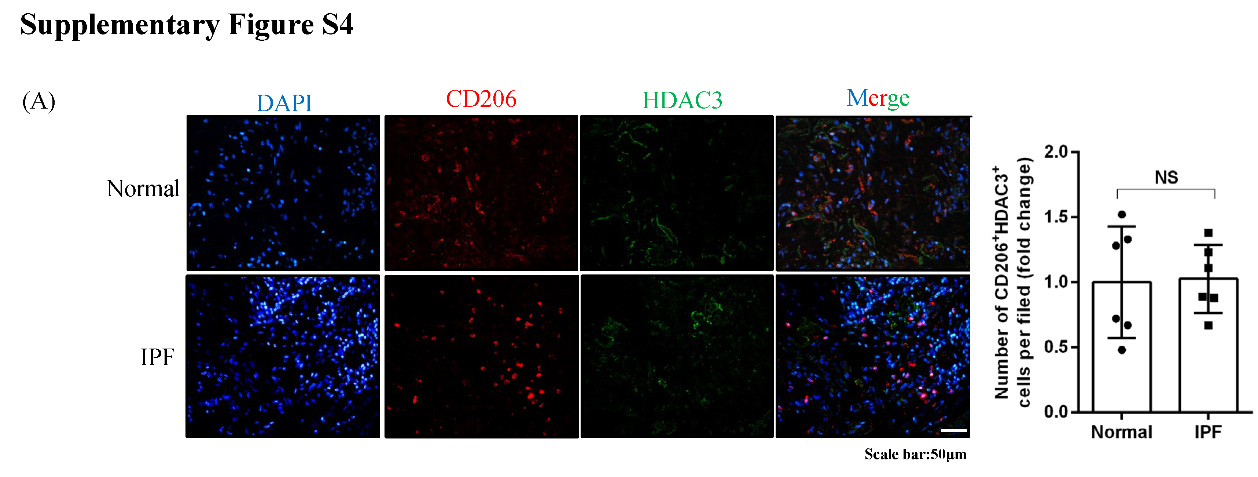


**Figure S4. (A).** Representative images of HDAC3 and CD206 immunofluorescence colocalization staining and quantitation in lung tissues from healthy controls and IPF patients. (×400, n=6). (**P* < 0.05, ***P* < 0.01, ****P* < 0.001, ^ns^P>0.05).





**Figure S5. (A).** The relative mRNA expression of AQP5, AGER, and HOPX in HDAC3-C and HDAC3-CKO mice in the presence of saline or BLM for 21d. (n=5). (**P* < 0.05, ***P* < 0.01, ****P* < 0.001, ^ns^P>0.05).


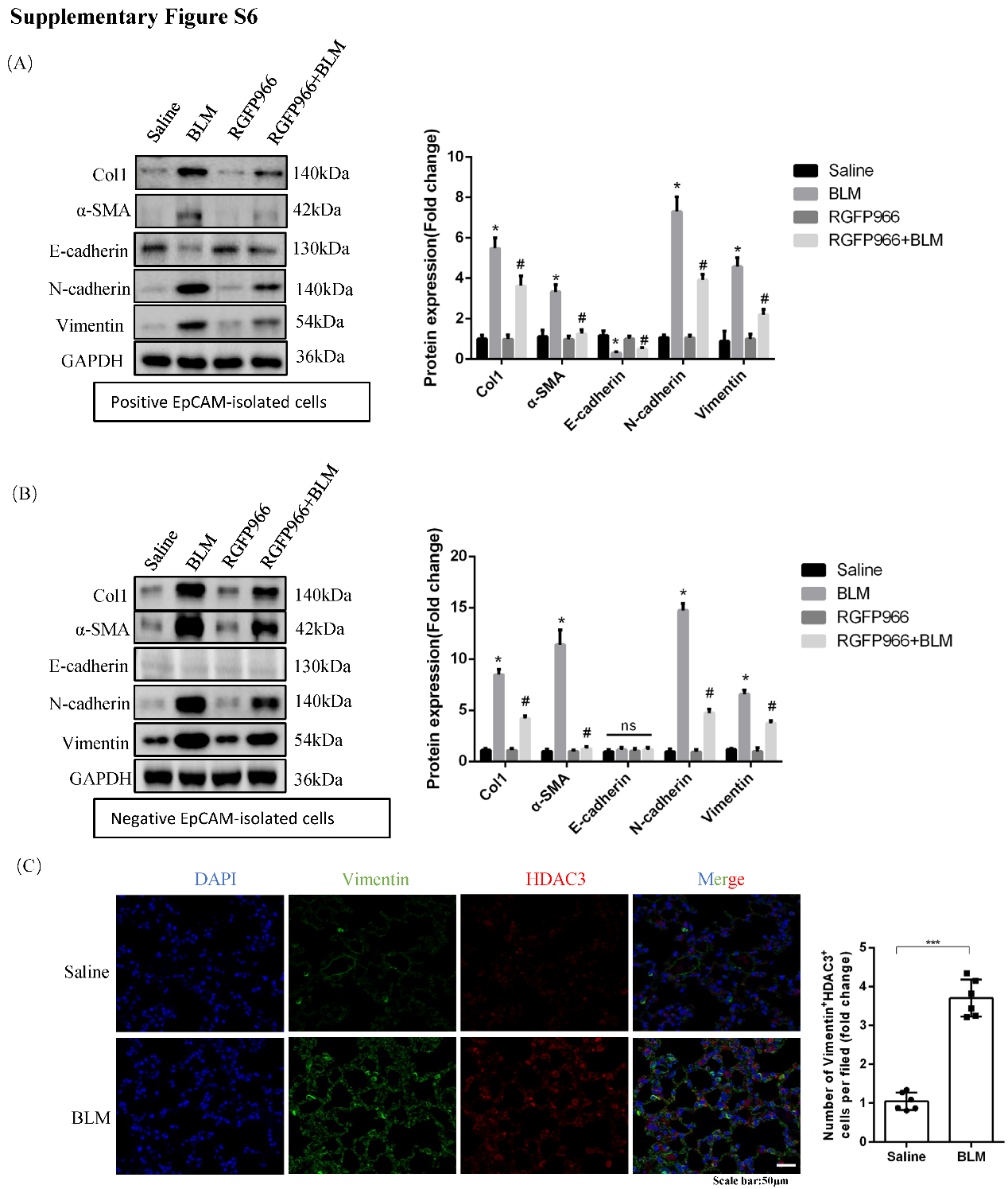


**Figure S6. (A).** The representative western blot images and the corresponding quantitative results of fibrotic markers (Col 1 and α-SMA), EMT markers (E-cadherin, N-cadherin and Vimentin) in positive EpCAM-isolated cells from lung tissues of BLM-treated mice at day 21 (n=5). **(B).** The representative western blot images and the corresponding quantitative results of fibrotic markers (Col 1 and α-SMA), EMT markers (E-cadherin, N-cadherin and Vimentin) in negative EpCAM-isolated cells from lung tissues of BLM-treated mice at day 21 (n=5). **(C).** Representative images of HDAC3 and Vimentin immunofluorescence colocalization staining and quantitation in lung tissues from BLM-treated mice at day 21 (×400, n=6) (**P* < 0.05 versus Saline group, ^#^*P* < 0.05 versus BLM group, ****P* < 0.001).


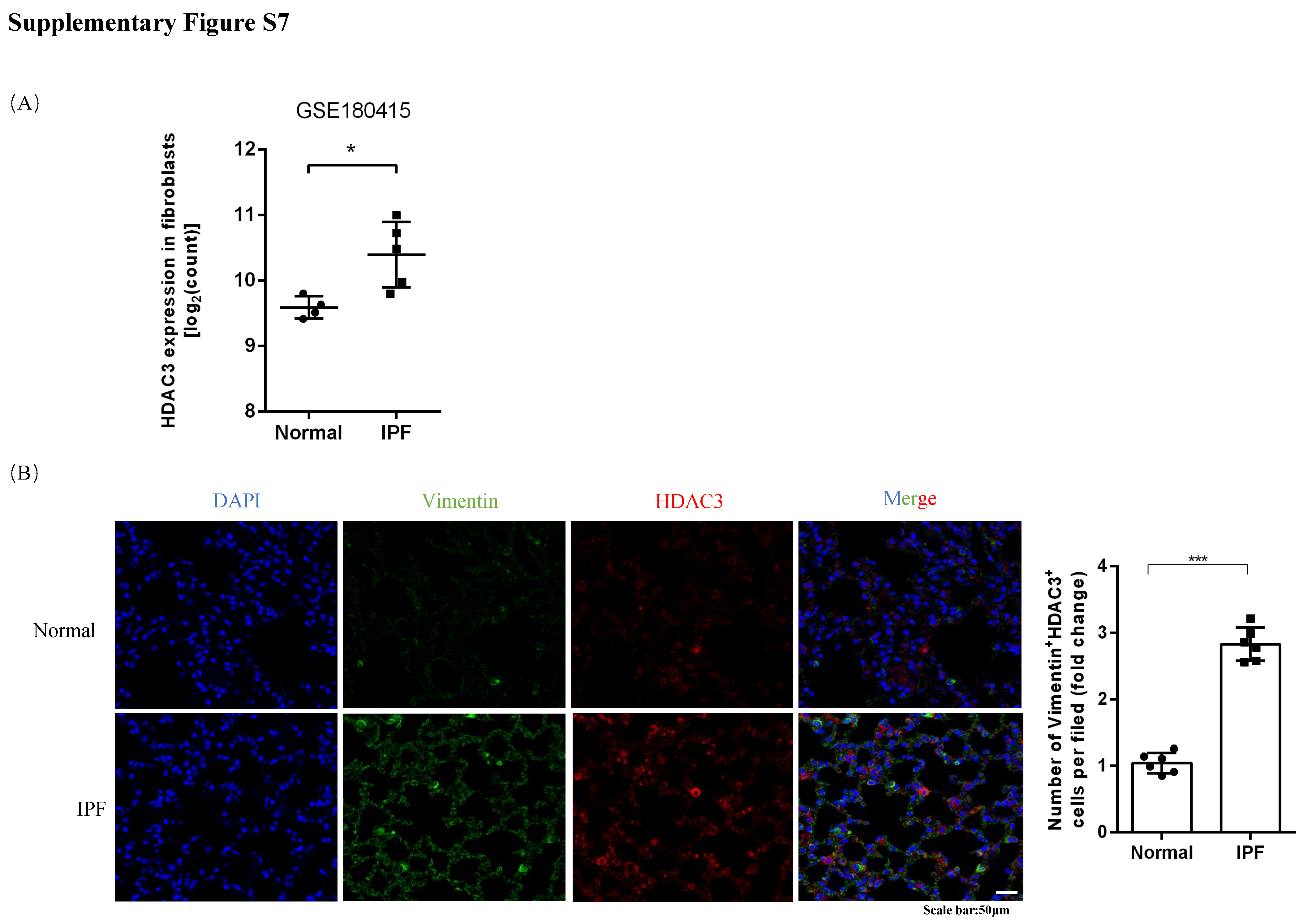


**Figure S7. (A).** Bioinformatics analysis of HDAC3 expression in fibroblasts from patients with IPF vs healthy controls. **(B).** Representative images of HDAC3 and Vimentin immunofluorescence colocalization staining and quantitation in lung tissues from patients with IPF and healthy controls (×400, n=6). (**P* < 0.05, ***P* < 0.01, ****P* < 0.001).


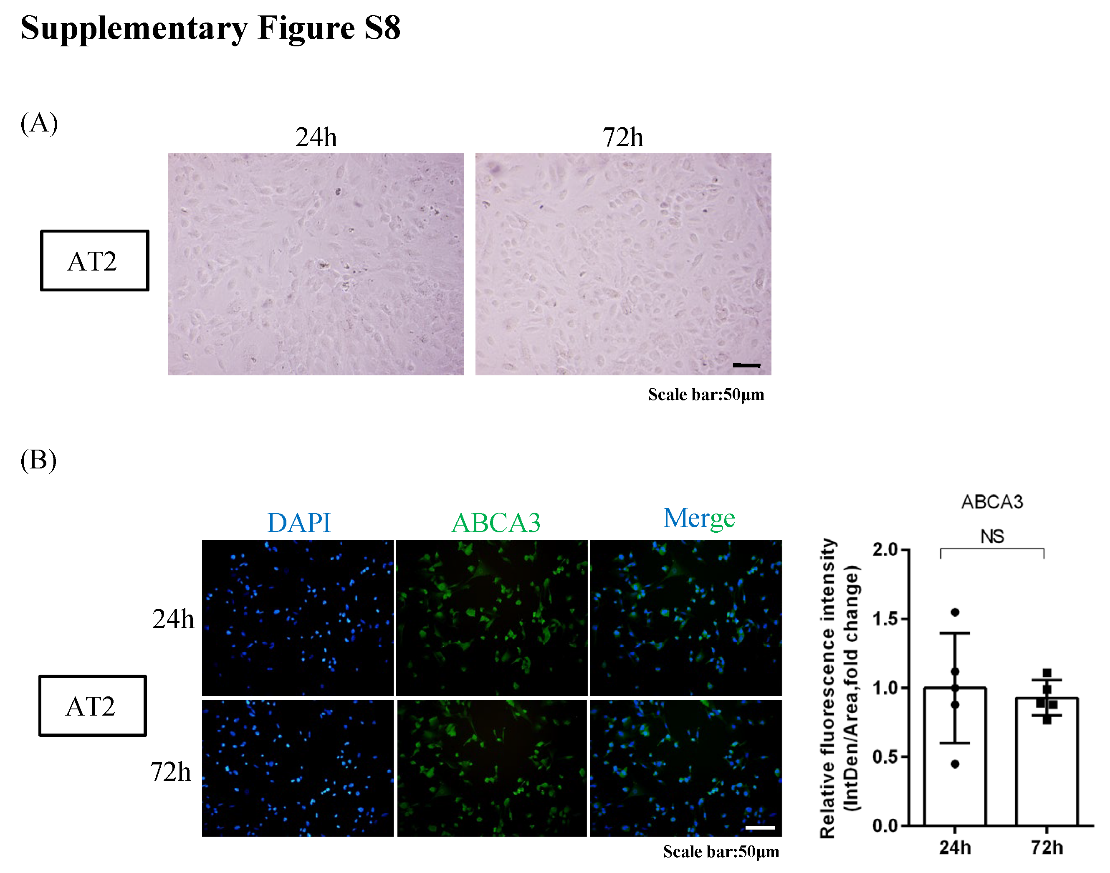


**Figure S8. (A).** Morphological results of primary mouse AT2 cells cultured for 24h and 72h (×200). There had no significant difference between the two groups. **(B).** Representative images of ABCA3 immunofluorescence staining and quantitation in AT2 cells after cultured for 24h and 72h. ABCA3, a specific marker of AT2 cells, had no significant change at 24h and 72h (×200, n=5). (**P* < 0.05, ***P* < 0.01, ****P* < 0.001, ^ns^P>0.05).
